# An Historical Overview: The Discovery of How NK Cells Can Kill Enemies, Recruit Defense Troops, and More

**DOI:** 10.3389/fimmu.2019.01415

**Published:** 2019-06-19

**Authors:** Massimo Vitale, Claudia Cantoni, Mariella Della Chiesa, Guido Ferlazzo, Simona Carlomagno, Daniela Pende, Michela Falco, Annamaria Pessino, Letizia Muccio, Andrea De Maria, Emanuela Marcenaro, Lorenzo Moretta, Simona Sivori

**Affiliations:** ^1^U.O.C. Immunologia, IRCCS Ospedale Policlinico San Martino, Genoa, Italy; ^2^Department of Experimental Medicine, University of Genoa, Genoa, Italy; ^3^Centre of Excellence for Biomedical Research, University of Genoa, Genoa, Italy; ^4^Laboratory of Clinical and Experimental Immunology, Integrated Department of Services and Laboratories, IRCCS Istituto Giannina Gaslini, Genoa, Italy; ^5^Laboratory of Immunology and Biotherapy, Department of Human Pathology, University of Messina, Messina, Italy; ^6^Medical Oncology Unit 1, IRCCS Ospedale Policlinico San Martino, Genoa, Italy; ^7^Dipartimento di Scienze della Salute (DISSAL), University of Genoa, Genoa, Italy; ^8^Clinica Malattie Infettive, IRCCS Ospedale Policlinico San Martino, Genoa, Italy; ^9^Laboratory of Tumor Immunology, Department of Immunology, IRCCS Ospedale Bambino Gesù, Rome, Italy

**Keywords:** human natural killer cells, innate immunity, natural cytotoxicity receptors, Toll-like receptors, activating NK receptors

## Abstract

Natural killer (NK) cells were originally defined as effector lymphocytes of innate immunity characterized by the unique ability of killing tumor and virally infected cells without any prior priming and expansion of specific clones. The “missing-self” theory, proposed by Klas Karre, the seminal discovery of the first prototypic HLA class I-specific inhibitory receptors, and, later, of the Natural Cytotoxicity Receptors (NCRs) by Alessandro Moretta, provided the bases to understand the puzzling behavior of NK cells. Actually, those discoveries proved crucial also for many of the achievements that, along the years, have contributed to the modern view of these cells. Indeed, NK cells, besides killing susceptible targets, are now known to functionally interact with different immune cells, sense pathogens using TLR, adapt their responses to the local environment, and, even, mount a sort of immunological memory. In this review, we will specifically focus on the main activating NK receptors and on their crucial role in the ever-increasing number of functions assigned to NK cells and other innate lymphoid cells (ILCs).

## Introduction

When Alessandro Moretta was appointed as Professor of Histology at the University of Genoa and started to set up a new lab and recruit people, including most of the authors of this review, the knowledge of how NK cells could exert their activity against tumors and viruses was very limited. The “missing-self” hypothesis had just been proposed by Karre and Ljunggren (1), but there was no idea on the molecular mechanisms by which NK cells could spare the “good” cells and kill the “bad” ones. Within <10 years, Moretta's lab generated a large number of monoclonal antibodies (mAbs) that allowed the identification and characterization of many key receptors, including, among many others, the first-discovered Killer Ig-like receptors (KIRs) (2–4) and the Natural Cytotoxicity Receptors (NCRs) (5). These discoveries provided the mechanistic explanation of the “missing-self” theory. Indeed, they showed that NK cells could kill target cells by integrating signals from activating and inhibitory receptors, by recognizing ligands on tumor or virus-infected cells and by sensing changes in HLA class I expression (6–9).

Later studies indicated that NK cells, besides “killing the enemies,” could also “incite the defense troops” by interacting with Dendritic Cells (DCs) to induce and polarize the adaptive immune response (10–12). A relevant role for given NK receptors newly identified by the Moretta's group, together with certain Toll-like receptors (TLRs), was found also in this context (13–16). This field was then further investigated, revealing the quite large net of interactions that NK cells can undertake with innate (granulocytes and macrophages) and adaptive immune cells, and even stromal and tumor cells (17–24).

After this early era of major discoveries, studies on NK cells increased exponentially, revealing an extraordinarily complex world, which now comprises a number of circulating or specialized tissue-resident NK cell subsets (25). Some studies also showed that NK cells can adapt their function to environmental changes or even maintain memory of certain viral infections (26–30). Moreover, many of the ligands for the activating NK receptors have now been identified and demonstrated to be variably expressed by tumor or virus-infected cells (31, 32). Much information have been added to the mechanisms that regulate the availability and function of NK cells within tumor tissues giving hints on the possible use of NK cells in the therapy of solid tumors (33–38). Finally, the extensive studies of the KIR repertoire and the “old” data on NK/DC interaction have posed the basis for a reliable exploitation of NK cells in hematopoietic stem cell transplantation (HSCT) to cure hematologic malignancies (39–42), while the new findings on the immune checkpoints regulating T and NK cell functions have reinforced the idea of blocking HLA class I-specific NK receptors to unleash the NK cell anti-tumor potential. In this context, human/humanized anti-KIR or anti-NKG2A mAbs or combinations of mAbs blocking NKG2A and the PD-1/PD-L axis are tested in animal models and clinics (33, 43–48).

**Figure 1:**
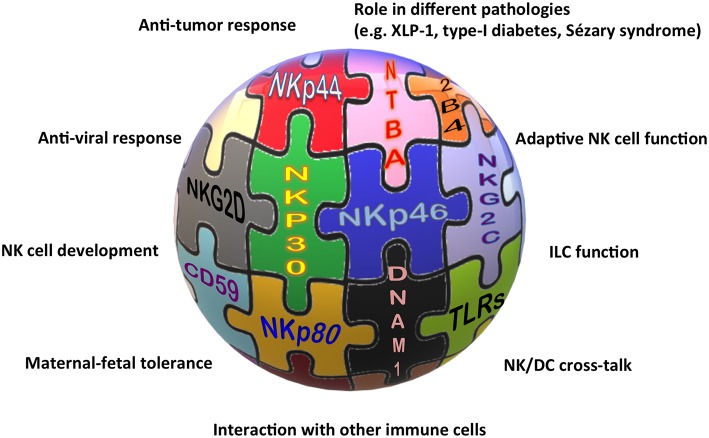
The “activating” solution of the NK cell puzzle. Different activating receptors collaborate to induce NK cell triggering in healthy and pathological conditions.

Alessandro Moretta, who has continued his work on NK cells with immutable enthusiasm all over his life, also contributed to these latter advances in the field with many key data, spanning from the tumor escape mechanisms acting on the activating receptor expression, to the characterization of the memory-like NK cell subset, the role of activating KIRs, and the role of immune checkpoints on NK cells in tumor patients. Nevertheless, it is indubitable that the identification of the first KIRs (which will be treated in a review aside) and of many NK activating receptors represents his real landmark discovery and legacy to Science. Indeed, the characterization of these receptors impressed an acceleration of the initial research and, still now, represents the basis for many new findings on NK cells and beyond (Figure 1).

The association of different NCR splice variants with tumor tissues or with non-pathological decidua tissues, the role of NKp30, NKp46, and NKp80 in the NK-mediated cross-talk with DCs, granulocytes, or monocytes, and the definition of NKp46 and NKp44 as markers of non-cytotoxic ILCs, are only some of the indications for the involvement of these receptors in near future studies on NK cell-based therapies against cancer, for long-standing investigations on the maternal-fetal tolerance, and, more extensively, on tissue homeostasis.

## Natural Cytotoxicity Receptors

Only few years after the identification of the first KIRs and of CD94/NKG2A, three non-HLA class I-specific activating receptors (namely NKp46, NKp30, and NKp44) were discovered in Alessandro Moretta's lab. These receptors, together with NKG2D, turned to be crucial for the recognition of both tumor and virus-infected cells (5, 49, 50). They were first characterized for their functional features (i.e., their ability to induce NK cell cytolytic activity and cytokine release) (51–54) and then also at the molecular level, when the cDNAs coding for these receptors were isolated (53, 55, 56) and the crystallographic structures were solved (57–60). NKp46, NKp30, and NKp44 were all selectively expressed on NK cells (although their expression was differently induced during activation) and revealed, since the initial studies, to be the main receptors responsible for the so-called “natural cytotoxicity” of NK cells. Thus, based on these findings, these receptors were collectively termed as Natural Cytotoxicity Receptors (NCRs), although neither the protein structure, nor the gene location gave indications for their belonging to a receptor family. Their discovery paved the way to a huge number of studies aimed at elucidating their function in both physiological and pathological conditions and characterizing the NCR/NCR ligand (NCR-L) interactions. As mentioned above, NCR expression was initially thought to be confined to NK cells, and NKp46 is still being considered a reliable NK cell-associated marker, both in humans and in mice (61, 62). Soon thereafter it became clear that these receptors could also be expressed in other immune cell types (63), extending their role to additional biological processes. For example, the characterization of the heterogeneous family of Innate Lymphoid Cells (ILCs) (25, 64, 65) revealed that NKp44 is also expressed by IFN-γ-producing intraepithelial ILC1 and by a subset of ILC3 present at the epithelial/mucosal surfaces, in tonsils, and in decidua tissue (66–71). Notably, NKp44^pos^ ILC3 display a unique cytokine pattern, being able to produce IL-22 following cytokine stimulation (68). In these cells, NKp44 triggering induces TNF-α production and activates a pro-inflammatory program (72), suggesting that NKp44 could play a role in the pathogenesis of different immune-mediated disease, including psoriasis (73). In addition, NCR^pos^ (NKp44^pos^) ILC3 have also been detected in the lymphoid infiltrate of non-small cell lung cancer, and have been found to release pro-inflammatory cytokines following interaction with tumor cells and tumor-associated fibroblasts (34, 67, 74). NKp46 expression has been detected in CD4^pos^ T lymphocytes derived from patients with Sézary syndrome, an aggressive form of cutaneous T-cell lymphoma (CTCL) (75). Notably, in these cells, NKp46 can act as an inhibitory co-receptor able to decrease CD3-mediated proliferation of Sézary cells, and has been proposed as an additional diagnostic marker, besides KIR3DL2, for the detection of these malignant cells (76).

One of the most investigated issues about NCRs is the characterization of their ligands. Although the landscape of NCR ligands is still incomplete, a common emerging theme is the multiplicity and heterogeneity of NCR/NCR-L interactions (31, 77–80). Most NCR ligands have been shown to activate NK cell function, while others dampen NK cell activation or act as “decoy ligands” when released in soluble form (81–85). The panel of cellular NCR-Ls currently includes surface glycoproteins, nuclear proteins that can be displayed at the cell surface, soluble molecules that can be either secreted, enzymatically shed, or conveyed through extracellular vesicles (82, 85–92). The expanding knowledge of NCR-Ls has opened the possibility of targeting NCR/NCR-L interactions in the context of cancer immunotherapy strategies. In addition, it has allowed the identification of several mechanisms of tumor escape related to the interaction between NK cells and malignant cells in the tumor microenvironment (22, 93–101). Finally, the importance of NK cell activity, and of NCRs in particular, in the therapeutic effect and outcome of oncolytic virotherapy has now being appreciated (102–104). NCR-Ls are also being studied as possible biomarkers in a variety of pathological conditions. Thus, a soluble form of B7-H6 (sB7-H6), an NKp30 ligand, has been demonstrated in the peritoneal fluid of ovarian cancer patients and in patients with metastatic gastrointestinal stromal tumor (GIST), neuroblastoma, or hepatocellular carcinoma (HCC) (83, 84, 105, 106). The presence of soluble BAG6/BAT3 (another NKp30-L) in the plasma of chronic lymphocytic leukemia patients was found to correlate with advanced disease stages (81). Along this line, high levels of soluble Nidogen-1, an NKp44 ligand, have been detected in the sera of patients with ovarian or lung cancer (107, 108).

Regarding the possibility of exploiting NCRs in anti-tumor approaches, it must be considered that NKp46 and NKp30 expression is down-regulated in NK cells derived from patients with different types of both hematological and non-hematological cancers (93, 109–116). This downmodulation leads to the impairment of NK cell anti-tumor potential and consequently to the need to develop strategies aimed at restoring NCR function (i.e., the use of cytokines, immunomodulatory drugs, anti-cancer drugs, or anti-KIR mAbs) (117–120). In addition, tumor cells themselves can become more resistant to NK cell-mediated attack by down-regulating NCR-Ls or releasing them in a soluble form (decoy ligands).

The role of NCRs stretches beyond cancer. B7-H6 is also involved in the inflammatory response: its expression is induced on monocytes following exposure to pro-inflammatory cytokines or TLR ligands, and high levels of sB7-H6 are found in the serum of patients with sepsis induced by Gram-negative bacteria (121). NK cells, in general, have been studied in different autoimmune disorders, including systemic lupus erythematosus, rheumatoid arthritis, multiple sclerosis, and type I diabetes (TID) (122–124). Focusing on NCRs, NKp46 has been shown to play a role in the pathogenesis of TID and in the destruction of normal pancreatic β cells (125), suggesting the possibility to target this receptor through specific anti-NKp46 mAbs (126).

A few years after the NCR discovery, the existence of different splice variants of these receptors was revealed (32, 127). Thus, three alternatively spliced NKp30 isoforms were identified, characterized by distinct intracellular regions and different functional capabilities. In GIST patients the prevalence of NKp30c isoform has been associated to decreased NK cell functionality and to reduced survival (128). Along this line, a similar pattern of NKp30 isoform expression has been detected in HCC patients (106). Notably, NKp30c isoform and sB7-H6 have been studied in metastatic GIST patients, revealing their possible use as predictive biomarkers of disease progression and response to imatinib mesylate treatment (105). NKp44 splice variants have been studied in different neoplastic disorders, and in particular in acute myeloid leukemia patients, indicating a correlation between the prevalence of the ITIM-bearing inhibitory NKp44-1 isoform and poor survival (129). The induction of NKp44-1 expression has been also observed in decidua NK cells, driven by cytokines released in the decidua microenvironment, and could play a role in promoting tolerance toward the fetus (127, 130).

Among the NCRs, NKp44 is the main receptor involved in the interplay between NK cells and trophoblast cells during pregnancy (131, 132), and is expressed also by a subset of ILC3 and by IFN-γ-producing ILC1-like cells found in the decidua (133). Decidua NK cells represent a peculiar NK cell subset, characterized by NKp44 expression, poor cytotoxic activity, and contributing to decidua development, vascularization, and tissue building/remodeling (134–136). Notably, in these cells, NKp44 triggering has been shown to induce IP10, IL-8, and VEGF release (132, 137).

## Activating Co-receptors

Alessandro Moretta gave fundamental contributions also to the identification and/or characterization of other surface receptors, including 2B4 (138–140), NTBA (141, 142), CD59 (143), and NKp80 (144), that play a complementary or a synergistic role with NCRs in inducing NK cell activation. Some of these molecules received great interest because of their involvement in NK cell function and development. 2B4 (145, 146) and NTBA (142), belonging to the signaling lymphocyte activation molecule (SLAM) family, have been shown to act as co-receptors, able to potentiate NK cell cytotoxic activity induced by the main triggering receptors, including NKp46 (140, 141). While 2B4 receptor recognizes CD48 (146, 147), NTBA is involved in homophilic interactions (142). Notably, 2B4 and NTBA dysfunction was described to be associated with a severe form of immunodeficiency, the X-linked lymphoproliferative syndrome type 1 (XLP-1), caused by mutations in *SH2D1A*, the gene encoding the signaling lymphocyte activation molecule (SLAM)-associated protein (SAP) (148). Interestingly, in the absence of SAP, the 2B4 and NTB-A co-receptors associate with the protein tyrosine phosphatases thus delivering inhibitory, instead of activating, signals (141, 149–151). This immune dysfunction is mainly responsible for the NK cell inability to kill EBV-infected B cells (B-EBV) that express CD48, resulting in extremely severe clinical consequences. A rapid diagnostic flowchart for XLP1, based on a 2B4-specific functional assay, combined with intra-cytoplasmic SAP staining, has been proposed (152). Moreover, the abnormal 2B4 function also influences 2B4 cross-talk with other NK receptors. Indeed, inhibitory 2B4 molecule selectively blocks ITAM-dependent activating receptors, namely NCR and CD16, while it affects neither NKG2D nor DNAM-1, which do not transduce through ITAM (152). This finding explains the selective inability, shown by NK cells, to kill B-EBV cells, which highly express CD48 and are mainly recognized by NCRs. In addition, in the NK cell repertoire of XLP-1 patients, NK cells lacking any self HLA class I-specific inhibitory receptor are highly represented and fully functional, indicating that the inhibitory 2B4 participates to NK cell education (153). Interestingly, a similar role for 2B4 has been described also in particular non-pathological processes. Indeed, at early stages of NK cell differentiation, when HLA class I-specific inhibitory receptors are not yet expressed, the delivery of inhibitory signals by 2B4, as a consequence of the late SAP expression, renders self-tolerant immature NK cells that otherwise would be autoreactive (154). Another peculiar situation is represented by decidua NK cells, in which 2B4 functions as an inhibitory receptor due to the absence or very low levels of SAP expression (155).

CD59 has been found to associate to NKp46 and NKp30 receptors and to enhance NK cell-mediated cytotoxic activity (143).

NKp80 molecule was initially described as a co-receptor, expressed by all NK cells, and able to cooperate with triggering receptors in the induction of natural cytotoxicity (144). Later, NKp80 was found to recognize the Activation-Induced C-type Lectin (AICL), a myeloid-specific activating receptor expressed by monocytes, macrophages, and granulocytes (156). NKp80-AICL interaction results in the secretion of pro-inflammatory cytokines from both cell types. In addition, it has been shown to participate in the NK cell-mediated elimination of malignant myeloid cells (156). NKp80 also plays an important role in the process of NK cell development. Indeed, it marks functionally mature NK cells developing in secondary lymphoid tissues (SLT). In particular, on the basis of NKp80 expression, two distinct subsets of SLT stage 4 cells can be distinguished: an NKp80^neg^ population with both NK- and ILC3-associated features and an NKp80^pos^ population with features similar to PB CD56^bright^ NK cells (157).

Among the surface molecules behaving as co-receptors in the activation of NK cell functions, a major role is assigned to DNAX Accessory Molecule (DNAM-1 or CD226), an adhesion molecule displaying activating function, expressed not only by all NK cells but also by T lymphocytes and monocytes (158). Alessandro Moretta's group gave an important contribution in this field with the identification of two different DNAM-1 ligands, namely PVR and Nectin-2, belonging to the Nectin family (159). These molecules are widely expressed on a variety of both hematological and solid tumors (160, 161), representing suitable targets for immunotherapeutic approaches (162). The role of DNAM-1 ligands in tumor cell recognition and killing by NK cells is actually more complex, since, besides DNAM-1, also the inhibitory receptors CD96 and TIGIT can recognize PVR or PVR and Nectin-2, respectively (163, 164). Accordingly, TIGIT and CD96 have been proposed as immune checkpoints, and are becoming appealing targets for the development of antibodies to be used in combination with other immune checkpoint inhibitors with the aim of unleashing both T and NK cell cytotoxic potential against tumors (165, 166).

## Role of NK Cells in Immune Regulation

### NK-DC Crosstalk

In the late ‘90s, it was becoming evident that innate immune cells do not act in isolation but potentiate their efficiency by interacting with each other, resulting even in the regulation of adaptive immune response. In 2001 Ralph Steinman (eventually a Nobel Laureate for the discovery of dendritic cells) visited our laboratories in Genoa and that occasion represented a starting point for a fruitful collaboration aimed at investigating the cross-talk occurring between human DCs and NK cells. As always, Prof. Moretta's insights were pivotal in all the studies carried out in that period, identifying which receptors and which subsets of these two innate immune components participate in this interaction, how this last one influences immune responses and to which extent similar stimuli (e.g., TLR ligands) are integrated by DCs and NK cells during innate immunity.

Until then, DCs were known for their critical role in initiating immune responses and priming antigen-specific T cell response (167), acting as sentinels in peripheral tissues, continuously sampling the environment. The dogma also foresaw that upon activation by danger signals, they up-regulated chemokine receptors and co-stimulatory molecules, which allowed them to migrate into lymph nodes and to efficiently induce T cell responses (167). Thus, the idea that DCs could also act as early activators of innate lymphocytes and, in turn, receive activating signals by activated NK cells, was ground-breaking in the field of innate immunity (14).

One of the relevant outcomes of NK/DC interaction is the so called “editing” of DCs, a term coined by Prof. Moretta to indicate the ability of NK cells to eliminate DCs in immature stage, and therefore *bona fide* tolerogenic DCs, while sparing activated/mature DCs able to efficiently induce the subsequent adaptive immune response in secondary lymphoid organs (12, 168, 169). The protective mechanisms of mature DCs was identified in the up-regulation of HLA class I molecules, especially of the non-classical HLA-E (170), occurring upon activation of DCs by either danger signals or NK cells themselves. At the same time, also the activating receptors involved in DC recognition by NK cells were identified (12, 171). The relevance of NKp30 receptor in NK/DC cross-talk was not limited to the mechanisms of killing of immature DCs but extended to the maturation process of DCs upon interaction with NK cells (172).

Remarkably, this cytolytic DC editing by NK cells was identified as a NK-mediated capability of dampening the graft-vs.-host disease in bone marrow transplantation (40) and graft rejection in solid organ transplantation (173, 174). It is noteworthy that, in case of improved skin graft rejection, NK cells were found to home to lymph nodes where they killed allogeneic DCs in a perforin-dependent manner (174).

Interestingly, and consistent with their concomitant role during the early phase of immune responses, NK cells and DCs are often able to sense similar stimuli in parallel. It was reported by Moretta's group that TLR engagement not only activates immature DCs but also renders NK cells more prone to receive triggering signals from pathogen-associated molecules, thus exerting a regulatory control on the early steps of innate immune responses against infectious agents (16), as more specifically addressed in the next paragraph.

All these studies on DC/NK interactions indicate a critical role for NK cells in the initiation and regulation of immune responses and provide a strong rationale for a combined targeting of NK cells and DCs in novel immunotherapeutic strategies, harnessing this cellular cross-talk in the treatment of patients with cancer and chronic infections resistant to conventional therapies.

Alessandro Moretta's contribution to the knowledge on the molecular basis of these cellular interactions paved the way to clinical interventions exploiting DC/NK cell cooperation. As a matter of fact, NK cell activation by DCs is particularly efficient, since DCs promote both effector functions and survival/proliferation of NK cells (169). As a whole, these basic discoveries, largely achieved under Prof. Moretta's guidance, revealed a particular translational relevance. For instance, in the field of haplo-HSCT, a beneficial role of NK cells in mediating graft-vs.-leukemia effects and in preventing GvHD was highlighted. The support provided by DCs for the proliferation/survival of NK cells is relevant also for establishing more efficient protocols for *ex vivo* NK cell expansion, given that NK cell-based immunotherapies are currently being reconsidered in both post-transplant hematological settings and in immunotherapy strategies for advanced solid tumors (41, 119, 175–180).

Finally, DCs activated by NK cells are better inducers of the anti-tumor CTL response, at least *in vitro*, as compared with the standard mature DCs currently employed in DC-based clinical trials (181) and could therefore be considered in immunization strategies for the development of next-generation vaccines (182, 183).

### Expression and Function of TLRs on Human NK Cells

Another field of research in which Prof. Moretta undoubtedly gave important contributions is the expression and function of TLRs in human NK cells. Indeed, in 2004 his group provided a solid experimental evidence that pathogen-associated products, known to strongly activate DCs and other innate immune cells, can also act on TLRs expressed by NK cells, inducing their activation both in terms of increased cytotoxicity and cytokine release (16). Alessandro Moretta and coworkers not only described the effect of TLR ligands on NK cell function, but also analyzed the role of TLR in the NK/DC crosstalk. This led to the concept of “NK cell-mediated editing of DCs,” the “quality control” process by which NK cells select DCs that are suited for T cell priming. The capability of TLR agonists of potentiating NK cell function was further defined in subsequent studies (184–193). Thus, in 2010 a peculiar cooperation between TLR9 and KIR3DL2 in inducing triggering of NK cell function upon treatment with CpG-ODN (TLR9 ligand) was described (194, 195). This study revealed that KIR3DL2 can bind CpG-ODNs at the NK cell surface and shuttle them to endosomes where TLR9 is localized, thus resulting in sharp down-regulation of KIR3DL2 surface expression and in TLR9-mediated induction of cytokine release. Moreover, it was demonstrated that the KIR Ig-domain involved in the direct recognition of CpG-ODN is represented by D0. Since this domain was hypothesized to be expressed by the putative ancestral mammalian KIR, these data suggested that, originally, certain KIRs could exert a function different from recognition of HLA class I molecules. Moreover, this newly defined functional capability of KIR3DL2 provided an important clue to understand the driving forces that led to the conservation of the KIR3DL2-encoding gene in all haplotypes, despite the low frequency, in the human population, of HLA-A^*^03 or -A^*^11 alleles (i.e., the ligands of KIR3DL2). Furthermore, in the Sézary Syndrome, in which KIR3DL2 represents a specific marker for the assessment of circulating tumor burden and for patient follow-up (76), CpG-ODN has been shown to promote not only the internalization of KIR3DL2 receptor but also the generation of apoptotic signals (196). Thus, CpG-ODN may exert a direct anti-tumor effect on Sézary cells through binding to KIR3DL2. In this context, a good clinical response without major side effects was observed upon class-B CpG-ODN subcutaneous administration in CTCL patients (197). CpG-DNA and other TLR agonists have been also explored as adjuvants for immunotherapy. Indeed, many clinical trials based on the use of CpG-ODNs as immunotherapeutic agents revealed that CpG-ODNs can promote Th1 immune responses and may be used in combination with chemotherapy to induce potent anti-tumor immune responses with relevant clinical benefits (186, 198, 199).

### NK Cell Subsets in Anti-virus Responses

Besides cancer and other diseases, NCRs also contribute to the NK cell-mediated control of viral infections through the recognition of virus-infected cells. Indeed, the first characterized NCR-Ls were of viral origin, namely influenza virus hemagglutinins (200, 201). Later on, additional viral ligands were identified and, in most cases, they were shown to induce NK cell activation following NCR engagement (31, 78, 202). It is of note, however, that some viral NCR-Ls can inhibit NCR functions, representing a possible immune evasion strategy (203). It has been very recently demonstrated in mouse that NK cells may play a regulatory role during acute and chronic lymphocytic choriomeningitis virus (LCMV) infection through the NKp46-mediated killing of LCMV-specific CD8 T cells (204).

In recent years Prof. Moretta and his co-workers gave major contributions to broaden our knowledge on NK cell diversity and functional specialization. This occurred primarily thanks to studies focused on NK cell-mediated responses to virus infections. Fundamental results came from the characterization of NK cells in patients chronically infected by HIV that revealed a deep functional impairment of NK cells likely determining their scarce capacity to efficiently control this virus. In this context, the relevance of NCR contribution to the course of HIV infection became clear when their reduced expression on NK cells in viraemic HIV-infected patients was demonstrated (205, 206). The NCR role in anti-viral response was also supported by the demonstration that NKp46 and NKp30 inducibility exerted a protective role in HIV-infected patients with excellent control not only of virus replication but, more importantly, also of retroviral reservoir (207, 208). Outside the HIV field, the study of NCR expression on NK cells similarly provided compelling evidence of their involvement in the response to acute HCV infection (209), and in HCV eradication in treated chronic carriers (210, 211). Interestingly, in chronically infected HIV patients the accumulation of a dysfunctional NK cell subset, virtually absent in healthy subjects, characterized by an aberrant CD56^neg^ CD16^bright^ surface signature (205, 212, 213) and defective DC editing was observed (214). This unusual population has been subsequently identified in several other pathological conditions including viral infections and immune deficiencies, in which these cells are responsible for an altered response to a chronic immune activation (215–219).

Besides HIV, a fundamental role in shaping NK cell repertoire and function has been described for CMV infection (220–222). Based on the pioneering studies by M. Lopez-Botet who first described the imprinting exerted by CMV on NK cells (223, 224), Alessandro Moretta contributed to identify CMV infection as a key driving force promoting the differentiation of functionally and phenotypically skewed NK cells with several studies conducted in HSCT recipients (225–228). In this setting, CMV infection/reactivation could induce not just NK cell maturation toward highly differentiated stages (characterized by the expression of CD94/NKG2C or activating KIRs), but also the unexpected acquisition of immunological memory. Indeed, NK cells maturing in CMV-reactivating patients share features with adaptive immune cells, such as long-term persistence, virus-induced clonal expansion, and epigenetic modifications (227, 229–234).

This anti-paradigmatic concept of memory or adaptive NK cells, to which Prof. Moretta contributed, holds important translational promise as this NK cell population characterized by longevity and superior ADCC ability, represents a potential tool for novel immunotherapeutic anti-cancer strategies, namely antibody-based tumor immunotherapies and generation of long-living anti-tumor CAR-NK cells (179, 235).

## NK Cell-Based Clinical Applications

**Figure 2:**
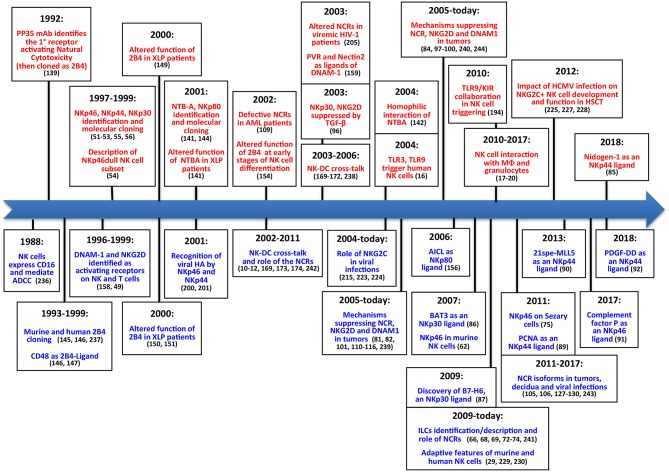
The main steps concerning activating NK receptors/coreceptors. The timeline illustrates the main discoveries concerning NK cell activating receptors during a timespan of about 30 years. Contributions deriving from Alessandro Moretta's research group are indicated in red **(upper part)**, while contributions obtained by other groups are shown in blue **(lower part)**.

**Figure 3:**
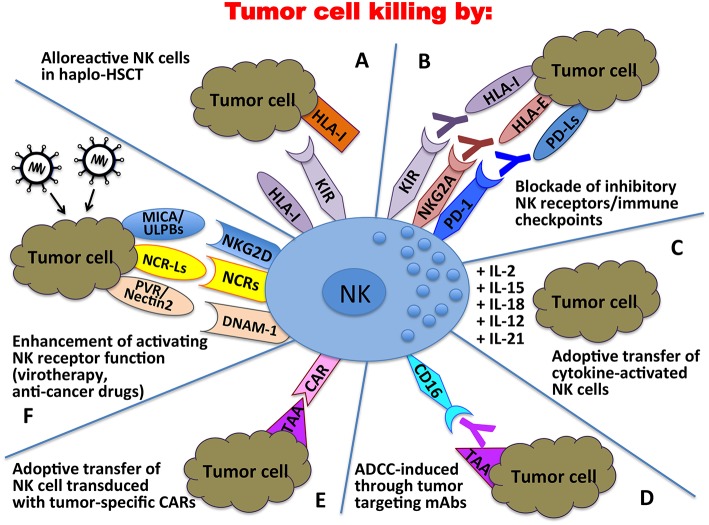
Clinical applications of NK cells in the immunotherapy against tumors. In haplo-HSCT, alloreactive NK cells can kill residual leukemic cells **(A)**; mAbs directed against immune checkpoints can unleash/restore NK cell anti-tumor activity **(B)**; tumor cell killing can be enhanced by adoptive transfer of cytokine-activated NK cells **(C)** or NK cells transduced with tumor-specific Chimeric Antigen Receptors (CARs) **(E)**; tumor targeting mAbs can induce NK cell-mediated ADCC **(D)**; activating NK receptor function can be potentiated through oncolytic virotherapy or the use of anti-cancer drugs **(F)**.

Altogether, these discoveries in the field of NK cell biology (Figure 2) (236–243) paved the way to the exploitation of these cells in different anti-tumor therapeutic approaches (Figure 3). Over the years important achievements have been obtained, and promising novel strategies have been designed. The most advanced clinical application exploiting the NK cell anti-tumor potential is in the field of haplo-identical HSCT (40–42, 235), in which donor-derived alloreactive NK cells (i.e., unable to recognize recipient HLA class I molecules) can exert a potent anti-leukemia effect. Moreover, the adoptive transfer of NK cells, in an autologous or allogeneic setting, can be pursued following NK cell activation and expansion with cytokines (118–120). The blockade of HLA class I-specific inhibitory receptors using human/humanized mAbs can be used to enhance killing of HLA class I^pos^ tumor cells. These mAbs can be used in combination with mAbs interfering with the PD-1/PD-L axis, as PD-1 can be expressed by human NK cells (46, 244). Another clinical approach is represented by the induction of ADCC against tumor cells by the use of antibodies specific for tumor-associated antigens (119).

More recently, the CAR technology, originally designed for T lymphocytes, has been applied also to NK cells, with promising results in the therapy of both hematological and solid tumors (118, 120). The ever-growing knowledge of activating NK receptor/ligand interactions is being applied in several strategies aimed to potentiate triggering signals through virotherapy or by the use of anti-cancer drugs capable of enhancing the expression of activating ligands on tumor cells and activating receptors on NK cells (102, 117). In conclusion, NK cell-based therapy used in combination with conventional therapeutic protocols could become more and more a powerful tool to be used in the cure of cancer.

## Concluding Remarks

By revisiting the discovery of the most important NK receptors and considering the technical approaches available at that time, one might have the impression that it has been simple to obtain those results. However, experienced researchers know that, actually, relevant pieces of information leading to a new discovery must be selected from an initially confusing, and often contradictory, mass of data. Alessandro had this ability, common to many gifted scientists, but he was also endowed with the uncommon talent of catching essential information and rendering simple what actually is very complex. We think that this has been the true and most important lesson for all of us and, undoubtedly, a major legacy for Immunology and Medicine.

## Author Contributions

All authors listed have made a substantial, direct and intellectual contribution to the work, and approved it for publication.

### Conflict of Interest Statement

The authors declare that the research was conducted in the absence of any commercial or financial relationships that could be construed as a potential conflict of interest.

## References

[1] Ljunggren HG Karre K. In search of the ‘missing self’: MHC molecules and NK cell recognition. Immunol Today. (1990) 11:237–44. 10.1016/0167-5699(90)90097-S 2201309

[2] Moretta A Bottino C Vitale M Pende D Biassoni R Mingari MC. Receptors for HLA class-I molecules in human natural killer cells. Annu Rev Immunol. (1996) 14:619–48. 10.1146/annurev.immunol.14.1.619 8717527

[3] Moretta A Biassoni R Bottino C Pende D Vitale M Poggi A. Major histocompatibility complex class I-specific receptors on human natural killer and T lymphocytes. Immunol Rev. (1997) 155:105–17. 10.1111/j.1600-065X.1997.tb00943.x 9059886

[4] Wagtmann N Rajagopalan S Winter CC Peruzzi M Long EO. Killer cell inhibitory receptors specific for HLA-C and HLA-B identified by direct binding and by functional transfer. Immunity. (1995) 3:801–9. 10.1016/1074-7613(95)90069-1 8777725

[5] Moretta A Bottino C Vitale M Pende D Cantoni C Mingari MC. Activating receptors and coreceptors involved in human natural killer cell-mediated cytolysis. Annu Rev Immunol. (2001) 19:197–223. 10.1146/annurev.immunol.19.1.197 11244035

[6] Long EO Burshtyn DN Clark WP Peruzzi M Rajagopalan S Rojo S. Killer cell inhibitory receptors: diversity, specificity, and function. Immunol Rev. (1997) 155:135–44. 10.1111/j.1600-065X.1997.tb00946.x 9059889

[7] Colonna M. Specificity and function of immunoglobulin superfamily NK cell inhibitory and stimulatory receptors. Immunol Rev. (1997) 155:127–33. 10.1111/j.1600-065X.1997.tb00945.x 9059888

[8] Parham P. MHC class I molecules and KIRs in human history, health and survival. Nat Rev Immunol. (2005) 5:201–14. 10.1038/nri1570 15719024

[9] Lanier LL. Natural killer cells: from no receptors to too many. Immunity. (1997) 6:371–8. 10.1016/S1074-7613(00)80280-0 9133416

[10] Piccioli D Sbrana S Melandri E Valiante NM. Contact-dependent stimulation and inhibition of dendritic cells by natural killer cells. J Exp Med. (2002) 195:335–41. 10.1084/jem.20010934 11828008 PMC2193592

[11] Gerosa F Baldani-Guerra B Nisii C Marchesini V Carra G Trinchieri G. Reciprocal activating interaction between natural killer cells and dendritic cells. J Exp Med. (2002) 195:327–33. 10.1084/jem.20010938 11828007 PMC2193595

[12] Ferlazzo G Tsang ML Moretta L Melioli G Steinman RM Munz C. Human dendritic cells activate resting natural killer (NK) cells and are recognized via the NKp30 receptor by activated NK cells. J Exp Med. (2002) 195:343–51. 10.1084/jem.20011149 11828009 PMC2193591

[13] Sivori S Carlomagno S Pesce S Moretta A Vitale M Marcenaro E. TLR/NCR/KIR: which one to use and when? Front Immunol. (2014) 5:105. 10.3389/fimmu.2014.00105 24678311 PMC3958761

[14] Moretta A. Natural killer cells and dendritic cells: rendezvous in abused tissues. Nat Rev Immunol. (2002) 2:957–64. 10.1038/nri956 12461568

[15] Della Chiesa M Sivori S Castriconi R Marcenaro E Moretta A. Pathogen-induced private conversations between natural killer and dendritic cells. Trends Microbiol. (2005) 13:128–36. 10.1016/j.tim.2005.01.006 15737731

[16] Sivori S Falco M Della Chiesa M Carlomagno S Vitale M Moretta L. CpG and double-stranded RNA trigger human NK cells by Toll-like receptors: induction of cytokine release and cytotoxicity against tumors and dendritic cells. Proc Natl Acad Sci USA. (2004) 101:10116–21. 10.1073/pnas.0403744101 15218108 PMC454174

[17] Thoren FB Riise RE Ousback J Della Chiesa M Alsterholm M Marcenaro E. Human NK Cells induce neutrophil apoptosis via an NKp46- and Fas-dependent mechanism. J Immunol. (2012) 188:1668–74. 10.4049/jimmunol.1102002 22231698

[18] Riise RE Bernson E Aurelius J Martner A Pesce S Della Chiesa M. TLR-stimulated neutrophils instruct NK cells to trigger dendritic cell maturation and promote adaptive T cell responses. J Immunol. (2015) 195:1121–8. 10.4049/jimmunol.1500709 26085684

[19] Bellora F Castriconi R Dondero A Reggiardo G Moretta L Mantovani A. The interaction of human natural killer cells with either unpolarized or polarized macrophages results in different functional outcomes. Proc Natl Acad Sci USA. (2010) 107:21659–64. 10.1073/pnas.1007654108 21118979 PMC3003022

[20] Pesce S Thoren FB Cantoni C Prato C Moretta L Moretta A. The innate immune cross talk between NK cells and eosinophils is regulated by the interaction of natural cytotoxicity receptors with eosinophil surface ligands. Front Immunol. (2017) 8:510. 10.3389/fimmu.2017.00510 28503177 PMC5408020

[21] Mattiola I Pesant M Tentorio PF Molgora M Marcenaro E Lugli E. Priming of human resting NK cells by autologous M1 macrophages via the engagement of IL-1beta, IFN-beta, and IL-15 Pathways. J Immunol. (2015) 195:2818–28. 10.4049/jimmunol.1500325 26276870

[22] Vitale M Cantoni C Pietra G Mingari MC Moretta L. Effect of tumor cells and tumor microenvironment on NK-cell function. Eur J Immunol. (2014) 44:1582–92. 10.1002/eji.201344272 24777896

[23] Martin-Fontecha A Thomsen LL Brett S Gerard C Lipp M Lanzavecchia A. Induced recruitment of NK cells to lymph nodes provides IFN-gamma for T(H)1 priming. Nat Immunol. (2004) 5:1260–5. 10.1038/ni1138 15531883

[24] Ardolino M Zingoni A Cerboni C Cecere F Soriani A Iannitto ML. DNAM-1 ligand expression on Ag-stimulated T lymphocytes is mediated by ROS-dependent activation of DNA-damage response: relevance for NK-T cell interaction. Blood. (2011) 117:4778–86. 10.1182/blood-2010-08-300954 21406724

[25] Freud AG Mundy-Bosse BL Yu J Caligiuri MA. The broad spectrum of human natural killer cell diversity. Immunity. (2017) 47:820–33. 10.1016/j.immuni.2017.10.008 29166586 PMC5728700

[26] Della Chiesa M Sivori S Carlomagno S Moretta L Moretta A. Activating KIRs and NKG2C in viral infections: toward NK cell memory? Front Immunol. (2015) 6:573. 10.3389/fimmu.2015.00573 26617607 PMC4638145

[27] Della Chiesa M Marcenaro E Sivori S Carlomagno S Pesce S Moretta A. Human NK cell response to pathogens. Semin Immunol. (2014) 26:152–60. 10.1016/j.smim.2014.02.001 24582551

[28] Muntasell A Vilches C Angulo A Lopez-Botet M. Adaptive reconfiguration of the human NK-cell compartment in response to cytomegalovirus: a different perspective of the host-pathogen interaction. Eur J Immunol. (2013) 43:1133–41. 10.1002/eji.201243117 23552990

[29] Sun JC Beilke JN Lanier LL. Adaptive immune features of natural killer cells. Nature. (2009) 457:557–61. 10.1038/nature07665 19136945 PMC2674434

[30] Vivier E Raulet DH Moretta A Caligiuri MA Zitvogel L Lanier LL Innate or adaptive immunity? The example of natural killer cells. Science. (2011) 331:44–9. 10.1126/science.1198687 21212348 PMC3089969

[31] Kruse PH Matta J Ugolini S Vivier E. Natural cytotoxicity receptors and their ligands. Immunol Cell Biol. (2014) 92:221–9. 10.1038/icb.2013.98 24366519

[32] Pazina T Shemesh A Brusilovsky M Porgador A Campbell KS. Regulation of the functions of natural cytotoxicity receptors by interactions with diverse ligands and alterations in splice variant expression. Front Immunol. (2017) 8:369. 10.3389/fimmu.2017.00369 28424697 PMC5371597

[33] Sivori S Vacca P Del Zotto G Munari E Mingari MC Moretta L. Human NK cells: surface receptors, inhibitory checkpoints, and translational applications. Cell Mol Immunol. (2019) 16:430–41. 10.1038/s41423-019-0206-4 30778167 PMC6474200

[34] Chiossone L Dumas PY Vienne M Vivier E Natural killer cells and other innate lymphoid cells in cancer. Nat Rev Immunol. (2018) 18:671–88. 10.1038/s41577-018-0061-z 30209347

[35] Pietra G Vitale C Pende D Bertaina A Moretta F Falco M. Human natural killer cells: news in the therapy of solid tumors and high-risk leukemias. Cancer Immunol Immunother. (2016) 65:465–76. 10.1007/s00262-015-1744-y 26289090 PMC11028670

[36] Tallerico R Garofalo C Carbone E. A new biological feature of natural killer cells: the recognition of solid tumor-derived cancer stem cells. Front Immunol. (2016) 7:179. 10.3389/fimmu.2016.00179 27242786 PMC4861715

[37] Stabile H Fionda C Gismondi A Santoni A. Role of distinct natural killer cell subsets in anticancer response. Front Immunol. (2017) 8:293. 10.3389/fimmu.2017.00293 28360915 PMC5352654

[38] Mehta RS Rezvani K. Chimeric antigen receptor expressing natural killer cells for the immunotherapy of cancer. Front Immunol. (2018) 9:283. 10.3389/fimmu.2018.00283 29497427 PMC5818392

[39] Moretta L Pietra G Montaldo E Vacca P Pende D Falco M. Human NK cells: from surface receptors to the therapy of leukemias and solid tumors. Front Immunol. (2014) 5:87. 10.3389/fimmu.2014.00087 24639677 PMC3945935

[40] Ruggeri L Capanni M Urbani E Perruccio K Shlomchik WD Tosti A. Effectiveness of donor natural killer cell alloreactivity in mismatched hematopoietic transplants. Science. (2002) 295:2097–100. 10.1126/science.1068440 11896281

[41] Pende D Marcenaro S Falco M Martini S Bernardo ME Montagna D. Anti-leukemia activity of alloreactive NK cells in KIR ligand-mismatched haploidentical HSCT for pediatric patients: evaluation of the functional role of activating KIR and redefinition of inhibitory KIR specificity. Blood. (2009) 113:3119–29. 10.1182/blood-2008-06-164103 18945967

[42] Cooley S Trachtenberg E Bergemann TL Saeteurn K Klein J Le CT. Donors with group B KIR haplotypes improve relapse-free survival after unrelated hematopoietic cell transplantation for acute myelogenous leukemia. Blood. (2009) 113:726–32. 10.1182/blood-2008-07-171926 18945962 PMC2628378

[43] Benson DM Jr. Bakan CE Zhang S Collins SM Liang J Srivastava S. IPH2101, a novel anti-inhibitory KIR antibody, and lenalidomide combine to enhance the natural killer cell versus multiple myeloma effect. Blood. (2011) 118:6387–91. 10.1182/blood-2011-06-360255 22031859 PMC3490103

[44] Pardoll DM. The blockade of immune checkpoints in cancer immunotherapy. Nat Rev Cancer. (2012) 12:252–64. 10.1038/nrc3239 22437870 PMC4856023

[45] Hughes PE Caenepeel S Wu LC. Targeted therapy and checkpoint immunotherapy combinations for the treatment of cancer. Trends Immunol. (2016) 37:462–76. 10.1016/j.it.2016.04.010 27216414

[46] Andre P Denis C Soulas C Bourbon-Caillet C Lopez J Arnoux T. Anti-NKG2A mAb Is a checkpoint inhibitor that promotes anti-tumor immunity by unleashing both T and NK cells. Cell. (2018) 175:1731–43 e13. 10.1016/j.cell.2018.10.014 30503213 PMC6292840

[47] Sanchez-Correa B Lopez-Sejas N Duran E Labella F Alonso C Solana R. Modulation of NK cells with checkpoint inhibitors in the context of cancer immunotherapy. Cancer Immunol Immunother. (2019) 68:861–70. 10.1007/s00262-019-02336-6 30953117 PMC11028212

[48] Morvan MG Lanier LL. NK cells and cancer: you can teach innate cells new tricks. Nat Rev Cancer. (2016) 16:7–19. 10.1038/nrc.2015.5 26694935

[49] Bauer S Groh V Wu J Steinle A Phillips JH Lanier LL. Activation of NK cells and T cells by NKG2D, a receptor for stress-inducible MICA. Science. (1999) 285:727–9. 10.1126/science.285.5428.727 10426993

[50] El-Gazzar A Groh V Spies T. Immunobiology and conflicting roles of the human NKG2D lymphocyte receptor and its ligands in cancer. J Immunol. (2013) 191:1509–15. 10.4049/jimmunol.1301071 23913973 PMC3736343

[51] Sivori S Vitale M Morelli L Sanseverino L Augugliaro R Bottino C. p46, a novel natural killer cell-specific surface molecule that mediates cell activation. J Exp Med. (1997) 186:1129–36. 10.1084/jem.186.7.1129 9314561 PMC2211712

[52] Vitale M Bottino C Sivori S Sanseverino L Castriconi R Marcenaro E. NKp44, a novel triggering surface molecule specifically expressed by activated natural killer cells, is involved in non-major histocompatibility complex-restricted tumor cell lysis. J Exp Med. (1998) 187:2065–72. 10.1084/jem.187.12.2065 9625766 PMC2212362

[53] Pende D Parolini S Pessino A Sivori S Augugliaro R Morelli L. Identification and molecular characterization of NKp30, a novel triggering receptor involved in natural cytotoxicity mediated by human natural killer cells. J Exp Med. (1999) 190:1505–16. 10.1084/jem.190.10.1505 10562324 PMC2195691

[54] Sivori S Pende D Bottino C Marcenaro E Pessino A Biassoni R NKp46 is the major triggering receptor involved in the natural cytotoxicity of fresh or cultured human NK cells. Correlation between surface density of NKp46 and natural cytotoxicity against autologous, allogeneic or xenogeneic target cells. Eur J Immunol. (1999) 29:1656–66. 10359120 10.1002/(SICI)1521-4141(199905)29:05<1656::AID-IMMU1656>3.0.CO;2-1

[55] Pessino A Sivori S Bottino C Malaspina A Morelli L Moretta L. Molecular cloning of NKp46: a novel member of the immunoglobulin superfamily involved in triggering of natural cytotoxicity. J Exp Med. (1998) 188:953–60. 10.1084/jem.188.5.953 9730896 PMC3207313

[56] Cantoni C Bottino C Vitale M Pessino A Augugliaro R Malaspina A. NKp44, a triggering receptor involved in tumor cell lysis by activated human natural killer cells, is a novel member of the immunoglobulin superfamily. J Exp Med. (1999) 189:787–96. 10.1084/jem.189.5.787 10049942 PMC2192947

[57] Cantoni C Ponassi M Biassoni R Conte R Spallarossa A Moretta A. The three-dimensional structure of the human NK cell receptor NKp44, a triggering partner in natural cytotoxicity. Structure. (2003) 11:725–34. 10.1016/S0969-2126(03)00095-9 12791260

[58] Ponassi M Cantoni C Biassoni R Conte R Spallarossa A Pesce A. Structure of the human NK cell triggering receptor NKp46 ectodomain. Biochem Biophys Res Commun. (2003) 309:317–23. 10.1016/j.bbrc.2003.08.007 12951052

[59] Li Y Wang Q Mariuzza RA. Structure of the human activating natural cytotoxicity receptor NKp30 bound to its tumor cell ligand B7-H6. J Exp Med. (2011) 208:703–14. 10.1084/jem.20102548 21422170 PMC3135353

[60] Joyce MG Tran P Zhuravleva MA Jaw J Colonna M Sun PD. Crystal structure of human natural cytotoxicity receptor NKp30 and identification of its ligand binding site. Proc Natl Acad Sci USA. (2011) 108:6223–8. 10.1073/pnas.1100622108 21444796 PMC3076882

[61] Biassoni R Pessino A Bottino C Pende D Moretta L Moretta A. The murine homologue of the human NKp46, a triggering receptor involved in the induction of natural cytotoxicity. Eur J Immunol. (1999) 29:1014–20. 10092106 10.1002/(SICI)1521-4141(199903)29:03<1014::AID-IMMU1014>3.0.CO;2-O

[62] Walzer T Blery M Chaix J Fuseri N Chasson L Robbins SH. Identification, activation, and selective *in vivo* ablation of mouse NK cells via NKp46. Proc Natl Acad Sci USA. (2007) 104:3384–9. 10.1073/pnas.0609692104 17360655 PMC1805551

[63] Hudspeth K Silva-Santos B Mavilio D. Natural cytotoxicity receptors: broader expression patterns and functions in innate and adaptive immune cells. Front Immunol. (2013) 4:69. 10.3389/fimmu.2013.00069 23518691 PMC3603285

[64] Vivier E Artis D Colonna M Diefenbach A Di Santo JP Eberl G. Innate lymphoid cells: 10 years on. Cell. (2018) 174:1054–66. 10.1016/j.cell.2018.07.017 30142344

[65] Vacca P Munari E Tumino N Moretta F Pietra G Vitale M Human natural killer cells and other innate lymphoid cells in cancer: friends or foes? Immunol Lett. (2018) 201:14–9. 10.1016/j.imlet.2018.11.004 30439479

[66] Fuchs A Vermi W Lee JS Lonardi S Gilfillan S Newberry RD. Intraepithelial type 1 innate lymphoid cells are a unique subset of IL-12- and IL-15-responsive IFN-gamma-producing cells. Immunity. (2013) 38:769–81. 10.1016/j.immuni.2013.02.010 23453631 PMC3634355

[67] Simoni Y Newell EW. Toward meaningful definitions of innate-lymphoid-cell subsets. Immunity. (2017) 46:760–1. 10.1016/j.immuni.2017.04.026 28514678

[68] Cella M Fuchs A Vermi W Facchetti F Otero K Lennerz JK. A human natural killer cell subset provides an innate source of IL-22 for mucosal immunity. Nature. (2009) 457:722–5. 10.1038/nature07537 18978771 PMC3772687

[69] Hoorweg K Peters CP Cornelissen F Aparicio-Domingo P Papazian N Kazemier G. Functional differences between human NKp44^−^ and NKp44^+^ RORC^+^ innate lymphoid cells. Front Immunol. (2012) 3:72. 10.3389/fimmu.2012.00072 22566953 PMC3342004

[70] Killig M Glatzer T Romagnani C. Recognition strategies of group 3 innate lymphoid cells. Front Immunol. (2014) 5:142. 10.3389/fimmu.2014.00142 24744763 PMC3978353

[71] Vacca P Vitale C Munari E Cassatella MA Mingari MC Moretta L. Human innate lymphoid cells: their functional and cellular interactions in decidua. Front Immunol. (2018) 9:1897. 10.3389/fimmu.2018.01897 30154799 PMC6102343

[72] Glatzer T Killig M Meisig J Ommert I Luetke-Eversloh M Babic M. RORγt^+^ innate lymphoid cells acquire a proinflammatory program upon engagement of the activating receptor NKp44. Immunity. (2013) 38:1223–35. 10.1016/j.immuni.2013.05.013 23791642

[73] Villanova F Flutter B Tosi I Grys K Sreeneebus H Perera GK. Characterization of innate lymphoid cells in human skin and blood demonstrates increase of NKp44+ ILC3 in psoriasis. J Invest Dermatol. (2014) 134:984–91. 10.1038/jid.2013.477 24352038 PMC3961476

[74] Carrega P Loiacono F Di Carlo E Scaramuccia A Mora M Conte R. NCR^+^ILC3 concentrate in human lung cancer and associate with intratumoral lymphoid structures. Nat Commun. (2015) 6:8280. 10.1038/ncomms9280 26395069

[75] Bensussan A Remtoula N Sivori S Bagot M Moretta A Marie-Cardine A. Expression and function of the natural cytotoxicity receptor NKp46 on circulating malignant CD4+ T lymphocytes of Sezary syndrome patients. J Invest Dermatol. (2011) 131:969–76. 10.1038/jid.2010.404 21191411

[76] Bagot M Moretta A Sivori S Biassoni R Cantoni C Bottino C. CD4+ cutaneous T-cell lymphoma cells express the p140-killer cell immunoglobulin-like receptor. Blood. (2001) 97:1388–91. 10.1182/blood.V97.5.1388 11222384

[77] Lam RA Chwee JY Le Bert N Sauer M Pogge von Strandmann E Gasser S. Regulation of self-ligands for activating natural killer cell receptors. Ann Med. (2013) 45:384–94. 10.3109/07853890.2013.792495 23701136

[78] Koch J Steinle A Watzl C Mandelboim O. Activating natural cytotoxicity receptors of natural killer cells in cancer and infection. Trends Immunol. (2013) 34:182–91. 10.1016/j.it.2013.01.003 23414611

[79] Brusilovsky M Radinsky O Yossef R Campbell KS Porgador A. Carbohydrate-mediated modulation of NK cell receptor function: structural and functional influences of heparan sulfate moieties expressed on NK cell surface. Front Oncol. (2014) 4:185. 10.3389/fonc.2014.00185 25077071 PMC4100077

[80] Horton NC Mathew PA. NKp44 and natural cytotoxicity receptors as damage-associated molecular pattern recognition receptors. Front Immunol. (2015) 6:31. 10.3389/fimmu.2015.00031 25699048 PMC4313717

[81] Reiners KS Topolar D Henke A Simhadri VR Kessler J Sauer M. Soluble ligands for NK cell receptors promote evasion of chronic lymphocytic leukemia cells from NK cell anti-tumor activity. Blood. (2013) 121:3658–65. 10.1182/blood-2013-01-476606 23509156 PMC3643764

[82] Schlecker E Fiegler N Arnold A Altevogt P Rose-John S Moldenhauer G. Metalloprotease-mediated tumor cell shedding of B7-H6, the ligand of the natural killer cell-activating receptor NKp30. Cancer Res. (2014) 74:3429–40. 10.1158/0008-5472.CAN-13-3017 24780758

[83] Semeraro M Rusakiewicz S Minard-Colin V Delahaye NF Enot D Vely F. Clinical impact of the NKp30/B7-H6 axis in high-risk neuroblastoma patients. Sci Transl Med. (2015) 7:283ra55. 10.1126/scitranslmed.aaa2327 25877893

[84] Pesce S Tabellini G Cantoni C Patrizi O Coltrini D Rampinelli F. B7-H6-mediated downregulation of NKp30 in NK cells contributes to ovarian carcinoma immune escape. Oncoimmunology. (2015) 4:e1001224. 10.1080/2162402X.2014.1001224 26137398 PMC4485754

[85] Gaggero S Bruschi M Petretto A Parodi M Zotto GD Lavarello C. Nidogen-1 is a novel extracellular ligand for the NKp44 activating receptor. Oncoimmunology. (2018) 7:e1470730. 10.1080/2162402X.2018.1470730 30228939 PMC6140582

[86] Pogge von Strandmann E Simhadri VR von Tresckow B Sasse S Reiners KS Hansen HP. Human leukocyte antigen-B-associated transcript 3 is released from tumor cells and engages the NKp30 receptor on natural killer cells. Immunity. (2007) 27:965–74. 10.1016/j.immuni.2007.10.010 18055229

[87] Brandt CS Baratin M Yi EC Kennedy J Gao Z Fox B. The B7 family member B7-H6 is a tumor cell ligand for the activating natural killer cell receptor NKp30 in humans. J Exp Med. (2009) 206:1495–503. 10.1084/jem.20090681 19528259 PMC2715080

[88] Hecht ML Rosental B Horlacher T Hershkovitz O De Paz JL Noti C. Natural cytotoxicity receptors NKp30, NKp44 and NKp46 bind to different heparan sulfate/heparin sequences. J Proteome Res. (2009) 8:712–20. 10.1021/pr800747c 19196184

[89] Rosental B Brusilovsky M Hadad U Oz D Appel MY Afergan F. Proliferating cell nuclear antigen is a novel inhibitory ligand for the natural cytotoxicity receptor NKp44. J Immunol. (2011) 187:5693–702. 10.4049/jimmunol.1102267 22021614 PMC3269963

[90] Baychelier F Sennepin A Ermonval M Dorgham K Debre P Vieillard V. Identification of a cellular ligand for the natural cytotoxicity receptor NKp44. Blood. (2013) 122:2935–42. 10.1182/blood-2013-03-489054 23958951

[91] Narni-Mancinelli E Gauthier L Baratin M Guia S Fenis A Deghmane AE. Complement factor P is a ligand for the natural killer cell-activating receptor NKp46. Sci Immunol. (2017) 2:eaam9628. 10.1126/sciimmunol.aam9628 28480349 PMC5419422

[92] Barrow AD Edeling MA Trifonov V Luo J Goyal P Bohl B. Natural killer cells control tumor growth by sensing a growth factor. Cell. (2018) 172:534–48 e19. 10.1016/j.cell.2017.11.037 29275861 PMC6684025

[93] Farnault L Sanchez C Baier C Le Treut T Costello RT. Hematological malignancies escape from NK cell innate immune surveillance: mechanisms and therapeutic implications. Clin Dev Immunol. (2012) 2012:421702. 10.1155/2012/421702 22899948 PMC3415262

[94] Stojanovic A Correia MP Cerwenka A. Shaping of NK cell responses by the tumor microenvironment. Cancer Microenviron. (2013) 6:135–46. 10.1007/s12307-012-0125-8 23242671 PMC3717064

[95] Baginska J Viry E Paggetti J Medves S Berchem G Moussay E. The critical role of the tumor microenvironment in shaping natural killer cell-mediated anti-tumor immunity. Front Immunol. (2013) 4:490. 10.3389/fimmu.2013.00490 24400010 PMC3872331

[96] Castriconi R Cantoni C Della Chiesa M Vitale M Marcenaro E Conte R. Transforming growth factor beta 1 inhibits expression of NKp30 and NKG2D receptors: consequences for the NK-mediated killing of dendritic cells. Proc Natl Acad Sci USA. (2003) 100:4120–5. 10.1073/pnas.0730640100 12646700 PMC153058

[97] Della Chiesa M Carlomagno S Frumento G Balsamo M Cantoni C Conte R. The tryptophan catabolite L-kynurenine inhibits the surface expression of NKp46- and NKG2D-activating receptors and regulates NK-cell function. Blood. (2006) 108:4118–25. 10.1182/blood-2006-03-006700 16902152

[98] Balsamo M Scordamaglia F Pietra G Manzini C Cantoni C Boitano M. Melanoma-associated fibroblasts modulate NK cell phenotype and antitumor cytotoxicity. Proc Natl Acad Sci USA. (2009) 106:20847–52. 10.1073/pnas.0906481106 19934056 PMC2791633

[99] Pietra G Manzini C Rivara S Vitale M Cantoni C Petretto A. Melanoma cells inhibit natural killer cell function by modulating the expression of activating receptors and cytolytic activity. Cancer Res. (2012) 72:1407–15. 10.1158/0008-5472.CAN-11-2544 22258454

[100] Huergo-Zapico L Parodi M Cantoni C Lavarello C Fernandez-Martinez JL Petretto A. NK-cell editing mediates epithelial-to-mesenchymal transition via phenotypic and proteomic changes in melanoma cell lines. Cancer Res. (2018) 78:3913–25. 10.1158/0008-5472.CAN-17-1891 29752261

[101] Park A Lee Y Kim MS Kang YJ Park YJ Jung H. Prostaglandin E2 secreted by thyroid cancer cells contributes to immune escape through the suppression of natural killer (NK) cell cytotoxicity and NK cell differentiation. Front Immunol. (2018) 9:1859. 10.3389/fimmu.2018.01859 30140269 PMC6094168

[102] Alvarez-Breckenridge CA Yu J Price R Wojton J Pradarelli J Mao H. NK cells impede glioblastoma virotherapy through NKp30 and NKp46 natural cytotoxicity receptors. Nat Med. (2012) 18:1827–34. 10.1038/nm.3013 23178246 PMC3668784

[103] Yoo JY Jaime-Ramirez AC Bolyard C Dai H Nallanagulagari T Wojton J. Bortezomib Treatment Sensitizes Oncolytic HSV-1-Treated Tumors to NK Cell Immunotherapy. Clin Cancer Res. (2016) 22:5265–76. 10.1158/1078-0432.CCR-16-1003 27390350 PMC5093037

[104] J GP Levesque S Workenhe ST Gujar S Le Boeuf F D RC Trial Watch: Oncolytic viro-immunotherapy of hematologic and solid tumors. Oncoimmunology. (2018) 7:e1503032 10.1080/2162402X.2018.1503032 30524901 PMC6279343

[105] Rusakiewicz S Perier A Semeraro M Pitt JM Pogge von Strandmann E Reiners KS. NKp30 isoforms and NKp30 ligands are predictive biomarkers of response to imatinib mesylate in metastatic GIST patients. Oncoimmunology. (2017) 6:e1137418. 10.1080/2162402X.2015.1137418 28197361 PMC5283614

[106] Mantovani S Oliviero B Lombardi A Varchetta S Mele D Sangiovanni A. Deficient natural killer cell NKp30-mediated function and altered NCR3 splice variants in hepatocellular carcinoma. Hepatology. (2019) 69:1165–79. 10.1002/hep.30235 30153337

[107] Li L Zhang Y Li N Feng L Yao H Zhang R. Nidogen-1: a candidate biomarker for ovarian serous cancer. Jpn J Clin Oncol. (2015) 45:176–82. 10.1093/jjco/hyu187 25378651

[108] Willumsen N Bager CL Leeming DJ Bay-Jensen AC Karsdal MA. Nidogen-1 degraded by Cathepsin S can be quantified in serum and is associated with non-small cell lung cancer. Neoplasia. (2017) 19:271–8. 10.1016/j.neo.2017.01.008 28282545 PMC5344320

[109] Costello RT Sivori S Marcenaro E Lafage-Pochitaloff M Mozziconacci MJ Reviron D. Defective expression and function of natural killer cell-triggering receptors in patients with acute myeloid leukemia. Blood. (2002) 99:3661–7. 10.1182/blood.V99.10.3661 11986221

[110] Fauriat C Just-Landi S Mallet F Arnoulet C Sainty D Olive D. Deficient expression of NCR in NK cells from acute myeloid leukemia: evolution during leukemia treatment and impact of leukemia cells in NCRdull phenotype induction. Blood. (2007) 109:323–30. 10.1182/blood-2005-08-027979 16940427

[111] Costello RT Knoblauch B Sanchez C Mercier D Le Treut T Sebahoun G. Expression of natural killer cell activating receptors in patients with chronic lymphocytic leukaemia. Immunology. (2012) 135:151–7. 10.1111/j.1365-2567.2011.03521.x 22044312 PMC3277717

[112] Platonova S Cherfils-Vicini J Damotte D Crozet L Vieillard V Validire P. Profound coordinated alterations of intratumoral NK cell phenotype and function in lung carcinoma. Cancer Res. (2011) 71:5412–22. 10.1158/0008-5472.CAN-10-4179 21708957

[113] Carrega P Morandi B Costa R Frumento G Forte G Altavilla G Natural killer cells infiltrating human nonsmall-cell lung cancer are enriched in CD56^bright^ CD16^−^ cells and display an impaired capability to kill tumor cells. Cancer. (2008) 112:863–75. 10.1002/cncr.23239 18203207

[114] Garcia-Iglesias T Del Toro-Arreola A Albarran-Somoza B Del Toro-Arreola S Sanchez-Hernandez PE Ramirez-Duenas MG. Low NKp30, NKp46 and NKG2D expression and reduced cytotoxic activity on NK cells in cervical cancer and precursor lesions. BMC Cancer. (2009) 9:186. 10.1186/1471-2407-9-186 19531227 PMC2704222

[115] Rocca YS Roberti MP Arriaga JM Amat M Bruno L Pampena MB. Altered phenotype in peripheral blood and tumor-associated NK cells from colorectal cancer patients. Innate Immun. (2013) 19:76–85. 10.1177/1753425912453187 22781631

[116] Pasero C Gravis G Guerin M Granjeaud S Thomassin-Piana J Rocchi P. Inherent and tumor-driven immune tolerance in the prostate microenvironment impairs natural killer cell antitumor activity. Cancer Res. (2016) 76:2153–65. 10.1158/0008-5472.CAN-15-1965 27197252

[117] Cifaldi L Locatelli F Marasco E Moretta L Pistoia V. Boosting natural killer cell-based immunotherapy with anticancer drugs: a perspective. Trends Mol Med. (2017) 23:1156–75. 10.1016/j.molmed.2017.10.002 29133133

[118] Guillerey C Huntington ND Smyth MJ. Targeting natural killer cells in cancer immunotherapy. Nat Immunol. (2016) 17:1025–36. 10.1038/ni.3518 27540992

[119] Chiossone L Vienne M Kerdiles YM Vivier E. Natural killer cell immunotherapies against cancer: checkpoint inhibitors and more. Semin Immunol. (2017) 31:55–63. 10.1016/j.smim.2017.08.003 28943093

[120] Souza-Fonseca-Guimaraes F Cursons J Huntington ND. The emergence of natural killer cells as a major target in cancer immunotherapy. Trends Immunol. (2019) 40:142–58. 10.1016/j.it.2018.12.003 30639050

[121] Matta J Baratin M Chiche L Forel JM Cognet C Thomas G. Induction of B7-H6, a ligand for the natural killer cell-activating receptor NKp30, in inflammatory conditions. Blood. (2013) 122:394–404. 10.1182/blood-2013-01-481705 23687088

[122] Flodstrom-Tullberg M Bryceson YT Shi FD Hoglund P Ljunggren HG. Natural killer cells in human autoimmunity. Curr Opin Immunol. (2009) 21:634–40. 10.1016/j.coi.2009.09.012 19892538

[123] Wensveen FM Jelencic V Valentic S Sestan M Wensveen TT Theurich S. NK cells link obesity-induced adipose stress to inflammation and insulin resistance. Nat Immunol. (2015) 16:376–85. 10.1038/ni.3120 25729921

[124] Gianchecchi E Delfino DV Fierabracci A. NK cells in autoimmune diseases: Linking innate and adaptive immune responses. Autoimmun Rev. (2018) 17:142–54. 10.1016/j.autrev.2017.11.018 29180124

[125] Gur C Porgador A Elboim M Gazit R Mizrahi S Stern-Ginossar N. The activating receptor NKp46 is essential for the development of type 1 diabetes. Nat Immunol. (2010) 11:121–8. 10.1038/ni.1834 20023661

[126] Berhani O Glasner A Kahlon S Duev-Cohen A Yamin R Horwitz E. Human anti-NKp46 antibody for studies of NKp46-dependent NK cell function and its applications for type 1 diabetes and cancer research. Eur J Immunol. (2019) 49:228–41. 10.1002/eji.201847611 30536875

[127] Siewiera J Gouilly J Hocine HR Cartron G Levy C Al-Daccak R. Natural cytotoxicity receptor splice variants orchestrate the distinct functions of human natural killer cell subtypes. Nat Commun. (2015) 6:10183. 10.1038/ncomms10183 26666685 PMC4682172

[128] Delahaye NF Rusakiewicz S Martins I Menard C Roux S Lyonnet L. Alternatively spliced NKp30 isoforms affect the prognosis of gastrointestinal stromal tumors. Nat Med. (2011) 17:700–7. 10.1038/nm.2366 21552268

[129] Shemesh A Brusilovsky M Hadad U Teltsh O Edri A Rubin E. Survival in acute myeloid leukemia is associated with NKp44 splice variants. Oncotarget. (2016) 7:32933–45. 10.18632/oncotarget.8782 27102296 PMC5078064

[130] Shemesh A Kugel A Steiner N Yezersky M Tirosh D Edri A. NKp44 and NKp30 splice variant profiles in decidua and tumor tissues: a comparative viewpoint. Oncotarget. (2016) 7:70912–23. 10.18632/oncotarget.12292 27765926 PMC5342598

[131] Sivori S Parolini S Marcenaro E Millo R Bottino C Moretta A. Triggering receptors involved in natural killer cell-mediated cytotoxicity against choriocarcinoma cell lines. Hum Immunol. (2000) 61:1055–8. 10.1016/S0198-8859(00)00201-9 11137207

[132] Vacca P Cantoni C Prato C Fulcheri E Moretta A Moretta L. Regulatory role of NKp44, NKp46, DNAM-1 and NKG2D receptors in the interaction between NK cells and trophoblast cells. Evidence for divergent functional profiles of decidual versus peripheral NK cells. Int Immunol. (2008) 20:1395–405. 10.1093/intimm/dxn105 18815119

[133] Vacca P Montaldo E Croxatto D Loiacono F Canegallo F Venturini PL. Identification of diverse innate lymphoid cells in human decidua. Mucosal Immunol. (2015) 8:254–64. 10.1038/mi.2014.63 25052762

[134] Moffett-King A. Natural killer cells and pregnancy. Nat Rev Immunol. (2002) 2:656–63. 10.1038/nri886 12209134

[135] Kopcow HD Allan DS Chen X Rybalov B Andzelm MM Ge B Human decidual NK cells form immature activating synapses and are not cytotoxic. Proc Natl Acad Sci USA. (2005) 102:15563–8. 10.1073/pnas.0507835102 16230631 PMC1266146

[136] Vacca P Chiossone L Mingari MC Moretta L. Heterogeneity of NK cells and other innate lymphoid cells in human and murine decidua. Front Immunol. (2019) 10:170. 10.3389/fimmu.2019.00170 30800126 PMC6375891

[137] Hanna J Goldman-Wohl D Hamani Y Avraham I Greenfield C Natanson-Yaron S. Decidual NK cells regulate key developmental processes at the human fetal-maternal interface. Nat Med. (2006) 12:1065–74. 10.1038/nm1452 16892062

[138] Tripodi G Poggi A Orengo AM Pella N Vitale M Sivori S Identification of a new surface molecule involved in the mechanism of cell to cell adhesion between human NK and tumor target cells. Cytotechnology. (1993) 11(Suppl 1):S109–11. 10.1007/BF00746071 7763735

[139] Moretta A Bottino C Tripodi G Vitale M Pende D Morelli L. Novel surface molecules involved in human NK cell activation and triggering of the lytic machinery. Int J Cancer Suppl. (1992) 7:6–10. 1428407

[140] Sivori S Parolini S Falco M Marcenaro E Biassoni R Bottino C. 2B4 functions as a co-receptor in human NK cell activation. Eur J Immunol. (2000) 30:787–93. 10.1002/1521-4141(200003)30:3<787::AID-IMMU787>3.0.CO;2-I 10741393

[141] Bottino C Falco M Parolini S Marcenaro E Augugliaro R Sivori S. NTB-A [correction of GNTB-A], a novel SH2D1A-associated surface molecule contributing to the inability of natural killer cells to kill Epstein-Barr virus-infected B cells in X-linked lymphoproliferative disease. J Exp Med. (2001) 194:235–46. 10.1084/jem.194.3.235 11489943 PMC2193462

[142] Falco M Marcenaro E Romeo E Bellora F Marras D Vely F. Homophilic interaction of NTBA, a member of the CD2 molecular family: induction of cytotoxicity and cytokine release in human NK cells. Eur J Immunol. (2004) 34:1663–72. 10.1002/eji.200424886 15162436

[143] Marcenaro E Augugliaro R Falco M Castriconi R Parolini S Sivori S. CD59 is physically and functionally associated with natural cytotoxicity receptors and activates human NK cell-mediated cytotoxicity. Eur J Immunol. (2003) 33:3367–76. 10.1002/eji.200324425 14635045

[144] Vitale M Falco M Castriconi R Parolini S Zambello R Semenzato G. Identification of NKp80, a novel triggering molecule expressed by human NK cells. Eur J Immunol. (2001) 31:233–42. 10.1002/1521-4141(200101)31:1<233::AID-IMMU233>3.0.CO;2-4 11265639

[145] Boles KS Nakajima H Colonna M Chuang SS Stepp SE Bennett M. Molecular characterization of a novel human natural killer cell receptor homologous to mouse 2B4. Tissue Antigens. (1999) 54:27–34. 10.1034/j.1399-0039.1999.540103.x 10458320

[146] Kubin MZ Parshley DL Din W Waugh JY Davis-Smith T Smith CA. Molecular cloning and biological characterization of NK cell activation-inducing ligand, a counterstructure for CD48. Eur J Immunol. (1999) 29:3466–77 10556801 10.1002/(SICI)1521-4141(199911)29:11<3466::AID-IMMU3466>3.0.CO;2-9

[147] Nakajima H Cella M Langen H Friedlein A Colonna M. Activating interactions in human NK cell recognition: the role of 2B4-CD48. Eur J Immunol. (1999) 29:1676–83. 10359122 10.1002/(SICI)1521-4141(199905)29:05<1676::AID-IMMU1676>3.0.CO;2-Y

[148] Sayos J Wu C Morra M Wang N Zhang X Allen D. The X-linked lymphoproliferative-disease gene product SAP regulates signals induced through the co-receptor SLAM. Nature. (1998) 395:462–9. 10.1038/26683 9774102

[149] Parolini S Bottino C Falco M Augugliaro R Giliani S Franceschini R. X-linked lymphoproliferative disease. 2B4 molecules displaying inhibitory rather than activating function are responsible for the inability of natural killer cells to kill Epstein-Barr virus-infected cells. J Exp Med. (2000) 192:337–46. 10.1084/jem.192.3.337 10934222 PMC2193227

[150] Nakajima H Cella M Bouchon A Grierson HL Lewis J Duckett CS. Patients with X-linked lymphoproliferative disease have a defect in 2B4 receptor-mediated NK cell cytotoxicity. Eur J Immunol. (2000) 30:3309–18. 10.1002/1521-4141(200011)30:11<3309::AID-IMMU3309>3.0.CO;2-3 11093147

[151] Tangye SG Phillips JH Lanier LL Nichols KE. Functional requirement for SAP in 2B4-mediated activation of human natural killer cells as revealed by the X-linked lymphoproliferative syndrome. J Immunol. (2000) 165:2932–6. 10.4049/jimmunol.165.6.2932 10975798

[152] Meazza R Tuberosa C Cetica V Falco M Loiacono F Parolini S XLP1 inhibitory effect by 2B4 does not affect DNAM-1 and NKG2D activating pathways in NK cells. Eur J Immunol. (2014) 44:1526–34. 10.1002/eji.201344312 24496997

[153] Meazza R Falco M Marcenaro S Loiacono F Canevali P Bellora F. Inhibitory 2B4 contributes to NK cell education and immunological derangements in XLP1 patients. Eur J Immunol. (2017) 47:1051–61. 10.1002/eji.201646885 28386908

[154] Sivori S Falco M Marcenaro E Parolini S Biassoni R Bottino C. Early expression of triggering receptors and regulatory role of 2B4 in human natural killer cell precursors undergoing *in vitro* differentiation. Proc Natl Acad Sci USA. (2002) 99:4526–31. 10.1073/pnas.072065999 11917118 PMC123681

[155] Vacca P Pietra G Falco M Romeo E Bottino C Bellora F. Analysis of natural killer cells isolated from human decidua: evidence that 2B4 (CD244) functions as an inhibitory receptor and blocks NK-cell function. Blood. (2006) 108:4078–85. 10.1182/blood-2006-04-017343 16931625

[156] Welte S Kuttruff S Waldhauer I Steinle A. Mutual activation of natural killer cells and monocytes mediated by NKp80-AICL interaction. Nat Immunol. (2006) 7:1334–42. 10.1038/ni1402 17057721

[157] Freud AG Keller KA Scoville SD Mundy-Bosse BL Cheng S Youssef Y. NKp80 defines a critical step during human natural killer cell development. Cell Rep. (2016) 16:379–91. 10.1016/j.celrep.2016.05.095 27373165 PMC4970225

[158] Shibuya A Campbell D Hannum C Yssel H Franz-Bacon K McClanahan T. DNAM-1, a novel adhesion molecule involved in the cytolytic function of T lymphocytes. Immunity. (1996) 4:573–81. 10.1016/S1074-7613(00)70060-4 8673704

[159] Bottino C Castriconi R Pende D Rivera P Nanni M Carnemolla B. Identification of PVR (CD155) and Nectin-2 (CD112) as cell surface ligands for the human DNAM-1 (CD226) activating molecule. J Exp Med. (2003) 198:557–67. 10.1084/jem.20030788 12913096 PMC2194180

[160] Pende D Spaggiari GM Marcenaro S Martini S Rivera P Capobianco A. Analysis of the receptor-ligand interactions in the natural killer-mediated lysis of freshly isolated myeloid or lymphoblastic leukemias: evidence for the involvement of the Poliovirus receptor (CD155) and Nectin-2 (CD112). Blood. (2005) 105:2066–73. 10.1182/blood-2004-09-3548 15536144

[161] Cerboni C Fionda C Soriani A Zingoni A Doria M Cippitelli M. The DNA damage response: a common pathway in the regulation of NKG2D and DNAM-1 ligand expression in normal, infected, and cancer cells. Front Immunol. (2014) 4:508. 10.3389/fimmu.2013.00508 24432022 PMC3882864

[162] Fionda C Soriani A Zingoni A Santoni A Cippitelli M. NKG2D and DNAM-1 ligands: molecular targets for NK cell-mediated immunotherapeutic intervention in multiple myeloma. Biomed Res Int. (2015) 2015:178698. 10.1155/2015/178698 26161387 PMC4486747

[163] Fuchs A Cella M Giurisato E Shaw AS Colonna M. Cutting edge: CD96 (tactile) promotes NK cell-target cell adhesion by interacting with the poliovirus receptor (CD155). J Immunol. (2004) 172:3994–8. 10.4049/jimmunol.172.7.3994 15034010

[164] Stanietsky N Simic H Arapovic J Toporik A Levy O Novik A. The interaction of TIGIT with PVR and PVRL2 inhibits human NK cell cytotoxicity. Proc Natl Acad Sci USA. (2009) 106:17858–63. 10.1073/pnas.0903474106 19815499 PMC2764881

[165] Dougall WC Kurtulus S Smyth MJ Anderson AC. TIGIT and CD96: new checkpoint receptor targets for cancer immunotherapy. Immunol Rev. (2017) 276:112–20. 10.1111/imr.12518 28258695

[166] Zhang Q Bi J Zheng X Chen Y Wang H Wu W. Blockade of the checkpoint receptor TIGIT prevents NK cell exhaustion and elicits potent anti-tumor immunity. Nat Immunol. (2018) 19:723–32. 10.1038/s41590-018-0132-0 29915296

[167] Banchereau J Steinman RM. Dendritic cells and the control of immunity. Nature. (1998) 392:245–52. 10.1038/32588 9521319

[168] Ferlazzo G Semino C Melioli G. HLA class I molecule expression is up-regulated during maturation of dendritic cells, protecting them from natural killer cell-mediated lysis. Immunol Lett. (2001) 76:37–41. 10.1016/S0165-2478(00)00323-0 11222911

[169] Ferlazzo G Pack M Thomas D Paludan C Schmid D Strowig T. Distinct roles of IL-12 and IL-15 in human natural killer cell activation by dendritic cells from secondary lymphoid organs. Proc Natl Acad Sci USA. (2004) 101:16606–11. 10.1073/pnas.0407522101 15536127 PMC534504

[170] Della Chiesa M Vitale M Carlomagno S Ferlazzo G Moretta L Moretta A. The natural killer cell-mediated killing of autologous dendritic cells is confined to a cell subset expressing CD94/NKG2A, but lacking inhibitory killer Ig-like receptors. Eur J Immunol. (2003) 33:1657–66. 10.1002/eji.200390042 12778484

[171] Pende D Castriconi R Romagnani P Spaggiari GM Marcenaro S Dondero A. Expression of the DNAM-1 ligands, Nectin-2 (CD112) and poliovirus receptor (CD155), on dendritic cells: relevance for natural killer-dendritic cell interaction. Blood. (2006) 107:2030–6. 10.1182/blood-2005-07-2696 16304049

[172] Vitale M Della Chiesa M Carlomagno S Pende D Arico M Moretta L. NK-dependent DC maturation is mediated by TNFalpha and IFNgamma released upon engagement of the NKp30 triggering receptor. Blood. (2005) 106:566–71. 10.1182/blood-2004-10-4035 15784725

[173] Yu G Xu X Vu MD Kilpatrick ED Li XC. NK cells promote transplant tolerance by killing donor antigen-presenting cells. J Exp Med. (2006) 203:1851–8. 10.1084/jem.20060603 16864660 PMC2118385

[174] Laffont S Seillet C Ortaldo J Coudert JD Guery JC. Natural killer cells recruited into lymph nodes inhibit alloreactive T-cell activation through perforin-mediated killing of donor allogeneic dendritic cells. Blood. (2008) 112:661–71. 10.1182/blood-2007-10-120089 18505782

[175] Terme M Ullrich E Delahaye NF Chaput N Zitvogel L. Natural killer cell-directed therapies: moving from unexpected results to successful strategies. Nat Immunol. (2008) 9:486–94. 10.1038/ni1580 18425105

[176] Barrow AD Colonna M. Tailoring Natural Killer cell immunotherapy to the tumour microenvironment. Semin Immunol. (2017) 31:30–6. 10.1016/j.smim.2017.09.001 28935344 PMC5659759

[177] Fang F Xiao W Tian Z. NK cell-based immunotherapy for cancer. Semin Immunol. (2017) 31:37–54. 10.1016/j.smim.2017.07.009 28838796

[178] Don Yun H Felices M Vallera DA Hinderlie P Cooley S Arock M. Trispecific killer engager CD16xIL15xCD33 potently induces NK cell activation and cytotoxicity against neoplastic mast cells. Blood Adv. (2018) 2:1580–4. 10.1182/bloodadvances.2018018176 29980573 PMC6039654

[179] Oei VYS Siernicka M Graczyk-Jarzynka A Hoel HJ Yang W Palacios D. Intrinsic functional potential of NK-cell subsets constrains retargeting driven by chimeric antigen receptors. Cancer Immunol Res. (2018) 6:467–80. 10.1158/2326-6066.CIR-17-0207 29459477

[180] Zhang C Oberoi P Oelsner S Waldmann A Lindner A Tonn T. Chimeric antigen receptor-engineered NK-92 cells: an off-the-shelf cellular therapeutic for targeted elimination of cancer cells and induction of protective antitumor immunity. Front Immunol. (2017) 8:533. 10.3389/fimmu.2017.00533 28572802 PMC5435757

[181] Kalinski P Nakamura Y Watchmaker P Giermasz A Muthuswamy R Mailliard RB. Helper roles of NK and CD8+ T cells in the induction of tumor immunity. Polarized dendritic cells as cancer vaccines. Immunol Res. (2006) 36:137–46. 10.1385/IR:36:1:137 17337774

[182] Ferlazzo G Munz C. Dendritic cell interactions with NK cells from different tissues. J Clin Immunol. (2009) 29:265–73. 10.1007/s10875-009-9283-y 19280325

[183] Rydyznski CE Waggoner SN. Boosting vaccine efficacy the natural (killer) way. Trends Immunol. (2015) 36:536–46. 10.1016/j.it.2015.07.004 26272882 PMC4567442

[184] Becker I Salaiza N Aguirre M Delgado J Carrillo-Carrasco N Kobeh LG. Leishmania lipophosphoglycan (LPG) activates NK cells through toll-like receptor-2. Mol Biochem Parasitol. (2003) 130:65–74. 10.1016/S0166-6851(03)00160-9 12946842

[185] Pisegna S Pirozzi G Piccoli M Frati L Santoni A Palmieri G. p38 MAPK activation controls the TLR3-mediated up-regulation of cytotoxicity and cytokine production in human NK cells. Blood. (2004) 104:4157–64. 10.1182/blood-2004-05-1860 15315972

[186] Schmidt KN Leung B Kwong M Zarember KA Satyal S Navas TA. APC-independent activation of NK cells by the Toll-like receptor 3 agonist double-stranded RNA. J Immunol. (2004) 172:138–43. 10.4049/jimmunol.172.1.138 14688319

[187] Marcenaro E Ferranti B Falco M Moretta L Moretta A. Human NK cells directly recognize Mycobacterium bovis via TLR2 and acquire the ability to kill monocyte-derived DC. Int Immunol. (2008) 20:1155–67. 10.1093/intimm/dxn073 18596023

[188] Chalifour A Jeannin P Gauchat JF Blaecke A Malissard M N'Guyen T. Direct bacterial protein PAMP recognition by human NK cells involves TLRs and triggers alpha-defensin production. Blood. (2004) 104:1778–83. 10.1182/blood-2003-08-2820 15166032

[189] Hart OM Athie-Morales V O'Connor GM Gardiner CM. TLR7/8-mediated activation of human NK cells results in accessory cell-dependent IFN-gamma production. J Immunol. (2005) 175:1636–42. 10.4049/jimmunol.175.3.1636 16034103

[190] Esin S Batoni G Pardini M Favilli F Bottai D Maisetta G. Functional characterization of human natural killer cells responding to *Mycobacterium bovis* bacille Calmette-Guerin. Immunology. (2004) 112:143–52. 10.1111/j.1365-2567.2004.01858.x 15096193 PMC1782452

[191] Alter G Suscovich TJ Teigen N Meier A Streeck H Brander C. Single-stranded RNA derived from HIV-1 serves as a potent activator of NK cells. J Immunol. (2007) 178:7658–66. 10.4049/jimmunol.178.12.7658 17548602

[192] Sivori S Carlomagno S Moretta L Moretta A. Comparison of different CpG oligodeoxynucleotide classes for their capability to stimulate human NK cells. Eur J Immunol. (2006) 36:961–7. 10.1002/eji.200535781 16525994

[193] Sivori S Falco M Carlomagno S Romeo E Moretta L Moretta A. Heterogeneity of TLR3 mRNA transcripts and responsiveness to poly (I:C) in human NK cells derived from different donors. Int Immunol. (2007) 19:1341–8. 10.1093/intimm/dxm105 17962643

[194] Sivori S Falco M Carlomagno S Romeo E Soldani C Bensussan A. A novel KIR-associated function: evidence that CpG DNA uptake and shuttling to early endosomes is mediated by KIR3DL2. Blood. (2010) 116:1637–47. 10.1182/blood-2009-12-256586 20147700

[195] Sivori S Falco M Moretta L Moretta A. Extending killer Ig-like receptor function: from HLA class I recognition to sensors of microbial products. Trends Immunol. (2010) 31:289–94. 10.1016/j.it.2010.05.007 20630802

[196] Ghazi B Thonnart N Bagot M Bensussan A Marie-Cardine A. KIR3DL2/CpG ODN interaction mediates Sezary syndrome malignant T cell apoptosis. J Invest Dermatol. (2015) 135:229–37. 10.1038/jid.2014.286 25007046

[197] Kim YH Girardi M Duvic M Kuzel T Link BK Pinter-Brown L. Phase I trial of a Toll-like receptor 9 agonist, PF-3512676 (CPG 7909), in patients with treatment-refractory, cutaneous T-cell lymphoma. J Am Acad Dermatol. (2010) 63:975–83. 10.1016/j.jaad.2009.12.052 20888065

[198] Matsumoto M Tatematsu M Nishikawa F Azuma M Ishii N Morii-Sakai A. Defined TLR3-specific adjuvant that induces NK and CTL activation without significant cytokine production *in vivo*. Nat Commun. (2015) 6:6280. 10.1038/ncomms7280 25692975

[199] Circelli L Tornesello M Buonaguro FM Buonaguro L. Use of adjuvants for immunotherapy. Hum Vaccin Immunother. (2017) 13:1774–7. 10.1080/21645515.2017.1321725 28604160 PMC5557248

[200] Mandelboim O Lieberman N Lev M Paul L Arnon TI Bushkin Y. Recognition of haemagglutinins on virus-infected cells by NKp46 activates lysis by human NK cells. Nature. (2001) 409:1055–60. 10.1038/35059110 11234016

[201] Arnon TI Lev M Katz G Chernobrov Y Porgador A Mandelboim O Recognition of viral hemagglutinins by NKp44 but not by NKp30. Eur J Immunol. (2001) 31:2680–9. 10.1002/1521-4141(200109)31:9<2680::AID-IMMU2680>3.0.CO;2-A 11536166

[202] Brusilovsky M Rosental B Shemesh A Appel MY Porgador A. Human NK cell recognition of target cells in the prism of natural cytotoxicity receptors and their ligands. J Immunotoxicol. (2012) 9:267–74. 10.3109/1547691X.2012.675366 22524686

[203] Arnon TI Achdout H Levi O Markel G Saleh N Katz G. Inhibition of the NKp30 activating receptor by pp65 of human cytomegalovirus. Nat Immunol. (2005) 6:515–23. 10.1038/ni1190 15821739

[204] Pallmer K Barnstorf I Baumann NS Borsa M Jonjic S Oxenius A. NK cells negatively regulate CD8 T cells via natural cytotoxicity receptor (NCR) 1 during LCMV infection. PLoS Pathog. (2019) 15:e1007725. 10.1371/journal.ppat.1007725 30995287 PMC6469806

[205] De Maria A Fogli M Costa P Murdaca G Puppo F Mavilio D. The impaired NK cell cytolytic function in viremic HIV-1 infection is associated with a reduced surface expression of natural cytotoxicity receptors (NKp46, NKp30 and NKp44). Eur J Immunol. (2003) 33:2410–8. 10.1002/eji.200324141 12938217

[206] Mavilio D Benjamin J Daucher M Lombardo G Kottilil S Planta MA. Natural killer cells in HIV-1 infection: dichotomous effects of viremia on inhibitory and activating receptors and their functional correlates. Proc Natl Acad Sci USA. (2003) 100:15011–6. 10.1073/pnas.2336091100 14645713 PMC299884

[207] Marras F Nicco E Bozzano F Di Biagio A Dentone C Pontali E Natural killer cells in HIV controller patients express an activated effector phenotype and do not up-regulate NKp44 on IL-2 stimulation. Proc Natl Acad Sci USA. (2013) 110:11970–5. 10.1073/pnas.1302090110 23818644 PMC3718138

[208] Marras F Casabianca A Bozzano F Ascierto ML Orlandi C Di Biagio A. Control of the HIV-1 DNA reservoir is associated *in vivo* and *in vitro* with NKp46/NKp30 (CD335 CD337) inducibility and interferon gamma production by transcriptionally unique NK cells. J Virol. (2017) 91:e00647–17. 10.1128/JVI.00647-17 28956765 PMC5686752

[209] Alter G Jost S Rihn S Reyor LL Nolan BE Ghebremichael M. Reduced frequencies of NKp30+NKp46+, CD161+, and NKG2D+ NK cells in acute HCV infection may predict viral clearance. J Hepatol. (2011) 55:278–88. 10.1016/j.jhep.2010.11.030 21168454 PMC3729214

[210] Bozzano F Picciotto A Costa P Marras F Fazio V Hirsch I. Activating NK cell receptor expression/function (NKp30, NKp46, DNAM-1) during chronic viraemic HCV infection is associated with the outcome of combined treatment. Eur J Immunol. (2011) 41:2905–14. 10.1002/eji.201041361 21695691

[211] Ascierto ML Bozzano F Bedognetti D Marras F Schechterly C Matsuura K. Inherent transcriptional signatures of NK cells are associated with response to IFNalpha + rivabirin therapy in patients with Hepatitis C Virus. J Transl Med. (2015) 13:77. 10.1186/s12967-015-0428-x 25849716 PMC4353456

[212] Fogli M Costa P Murdaca G Setti M Mingari MC Moretta L. Significant NK cell activation associated with decreased cytolytic function in peripheral blood of HIV-1-infected patients. Eur J Immunol. (2004) 34:2313–21. 10.1002/eji.200425251 15259029

[213] Mavilio D Lombardo G Benjamin J Kim D Follman D Marcenaro E. Characterization of CD56-/CD16+ natural killer (NK) cells: a highly dysfunctional NK subset expanded in HIV-infected viremic individuals. Proc Natl Acad Sci USA. (2005) 102:2886–91. 10.1073/pnas.0409872102 15699323 PMC549494

[214] Mavilio D Lombardo G Kinter A Fogli M La Sala A Ortolano S. Characterization of the defective interaction between a subset of natural killer cells and dendritic cells in HIV-1 infection. J Exp Med. (2006) 203:2339–50. 10.1084/jem.20060894 17000867 PMC2118111

[215] Farnault L Chambost H Michel G Thuret I de Saint Basile G Fischer A. Persistence of natural killer cells with expansion of a hypofunctional CD56-CD16+KIR+NKG2C+ subset in a patient with atypical Janus kinase 3-deficient severe combined immunodeficiency. J Allergy Clin Immunol. (2013) 131:1230–3. 10.1016/j.jaci.2012.08.047 23069490

[216] Bjorkstrom NK Ljunggren HG Sandberg JK. CD56 negative NK cells: origin, function, and role in chronic viral disease. Trends Immunol. (2010) 31:401–6. 10.1016/j.it.2010.08.003 20829113

[217] Gonzalez VD Falconer K Bjorkstrom NK Blom KG Weiland O Ljunggren HG. Expansion of functionally skewed CD56-negative NK cells in chronic hepatitis C virus infection: correlation with outcome of pegylated IFN-alpha and ribavirin treatment. J Immunol. (2009) 183:6612–8. 10.4049/jimmunol.0901437 19846870

[218] Bjorkstrom NK Lindgren T Stoltz M Fauriat C Braun M Evander M. Rapid expansion and long-term persistence of elevated NK cell numbers in humans infected with hantavirus. J Exp Med. (2011) 208:13–21. 10.1084/jem.20100762 21173105 PMC3023129

[219] Schlums H Jung M Han H Theorell J Bigley V Chiang SC. Adaptive NK cells can persist in patients with GATA2 mutation depleted of stem and progenitor cells. Blood. (2017) 129:1927–39. 10.1182/blood-2016-08-734236 28209719 PMC5383869

[220] Lopez-Botet M Muntasell A Vilches C. The CD94/NKG2C+ NK-cell subset on the edge of innate and adaptive immunity to human cytomegalovirus infection. Semin Immunol. (2014) 26:145–51. 10.1016/j.smim.2014.03.002 24666761

[221] Tesi B Schlums H Cichocki F Bryceson YT. Epigenetic regulation of adaptive NK cell diversification. Trends Immunol. (2016) 37:451–61. 10.1016/j.it.2016.04.006 27160662

[222] Rolle A Brodin P. Immune adaptation to environmental influence: the case of NK cells and HCMV. Trends Immunol. (2016) 37:233–43. 10.1016/j.it.2016.01.005 26869205

[223] Guma M Angulo A Vilches C Gomez-Lozano N Malats N Lopez-Botet M. Imprint of human cytomegalovirus infection on the NK cell receptor repertoire. Blood. (2004) 104:3664–71. 10.1182/blood-2004-05-2058 15304389

[224] Guma M Budt M Saez A Brckalo T Hengel H Angulo A. Expansion of CD94/NKG2C+ NK cells in response to human cytomegalovirus-infected fibroblasts. Blood. (2006) 107:3624–31. 10.1182/blood-2005-09-3682 16384928

[225] Della Chiesa M Falco M Podesta M Locatelli F Moretta L Frassoni F. Phenotypic and functional heterogeneity of human NK cells developing after umbilical cord blood transplantation: a role for human cytomegalovirus? Blood. (2012) 119:399–410. 10.1182/blood-2011-08-372003 22096237

[226] Della Chiesa M Muccio L Moretta A. CMV induces rapid NK cell maturation in HSCT recipients. Immunol Lett. (2013) 155:11–3. 10.1016/j.imlet.2013.09.020 24076315

[227] Muccio L Bertaina A Falco M Pende D Meazza R Lopez-Botet M. Analysis of memory-like natural killer cells in human cytomegalovirus-infected children undergoing alphabeta+T and B cell-depleted hematopoietic stem cell transplantation for hematological malignancies. Haematologica. (2016) 101:371–81. 10.3324/haematol.2015.134155 26659918 PMC4815729

[228] Muccio L Falco M Bertaina A Locatelli F Frassoni F Sivori S Late development of fcepsilonrgamma(neg) adaptive natural killer cells upon human cytomegalovirus reactivation in umbilical cord blood transplantation recipients. Front Immunol. (2018) 9:1050 10.3389/fimmu.2018.01050 29868012 PMC5968376

[229] Foley B Cooley S Verneris MR Pitt M Curtsinger J Luo X. Cytomegalovirus reactivation after allogeneic transplantation promotes a lasting increase in educated NKG2C+ natural killer cells with potent function. Blood. (2012) 119:2665–74. 10.1182/blood-2011-10-386995 22180440 PMC3311280

[230] Foley B Cooley S Verneris MR Curtsinger J Luo X Waller EK. Human cytomegalovirus (CMV)-induced memory-like NKG2C(+) NK cells are transplantable and expand *in vivo* in response to recipient CMV antigen. J Immunol. (2012) 189:5082–8. 10.4049/jimmunol.1201964 23077239 PMC3490031

[231] Schlums H Cichocki F Tesi B Theorell J Beziat V Holmes TD. Cytomegalovirus infection drives adaptive epigenetic diversification of NK cells with altered signaling and effector function. Immunity. (2015) 42:443–56. 10.1016/j.immuni.2015.02.008 25786176 PMC4612277

[232] Lee J Zhang T Hwang I Kim A Nitschke L Kim M. Epigenetic modification and antibody-dependent expansion of memory-like NK cells in human cytomegalovirus-infected individuals. Immunity. (2015) 42:431–42. 10.1016/j.immuni.2015.02.013 25786175 PMC4537797

[233] Cichocki F Taras E Chiuppesi F Wagner JE Blazar BR Brunstein C. Adaptive NK cell reconstitution is associated with better clinical outcomes. JCI insight. (2019) 4:125553. 10.1172/jci.insight.125553 30674718 PMC6413795

[234] Hammer Q Ruckert T Borst EM Dunst J Haubner A Durek P. Peptide-specific recognition of human cytomegalovirus strains controls adaptive natural killer cells. Nat Immunol. (2018) 19:453–63. 10.1038/s41590-018-0082-6 29632329

[235] Locatelli F Pende D Falco M Della Chiesa M Moretta A Moretta L. NK Cells mediate a crucial graft-versus-leukemia effect in haploidentical-HSCT to cure high-risk acute leukemia. Trends Immunol. (2018) 39:577–90. 10.1016/j.it.2018.04.009 29793748

[236] Lanier LL Ruitenberg JJ Phillips JH. Functional and biochemical analysis of CD16 antigen on natural killer cells and granulocytes. J Immunol. (1988) 141:3478–85. 2903193

[237] Mathew PA Garni-Wagner BA Land K Takashima A Stoneman E Bennett M. Cloning and characterization of the 2B4 gene encoding a molecule associated with non-MHC-restricted killing mediated by activated natural killer cells and T cells. J Immunol. (1993) 151:5328–37. 8228228

[238] Vitale M Della Chiesa M Carlomagno S Romagnani C Thiel A Moretta L. The small subset of CD56brightCD16- natural killer cells is selectively responsible for both cell proliferation and interferon-gamma production upon interaction with dendritic cells. Eur J Immunol. (2004) 34:1715–22. 10.1002/eji.200425100 15162442

[239] Ghiringhelli F Menard C Terme M Flament C Taieb J Chaput N. CD4+CD25+ regulatory T cells inhibit natural killer cell functions in a transforming growth factor-beta-dependent manner. J Exp Med. (2005) 202:1075–85. 10.1084/jem.20051511 16230475 PMC2213209

[240] Marcenaro E Della Chiesa M Bellora F Parolini S Millo R Moretta L IL-12 or IL-4 prime human NK cells to mediate functionally divergent interactions with dendritic cells or tumors. J Immunol. (2005) 174:3992–8. 10.4049/jimmunol.174.7.3992 15778356

[241] Sanos SL Bui VL Mortha A Oberle K Heners C Johner C. RORgammat and commensal microflora are required for the differentiation of mucosal interleukin 22-producing NKp46+ cells. Nat Immunol. (2009) 10:83–91. 10.1038/ni.1684 19029903 PMC4217274

[242] Magri G Muntasell A Romo N Saez-Borderias A Pende D Geraghty DE. NKp46 and DNAM-1 NK-cell receptors drive the response to human cytomegalovirus-infected myeloid dendritic cells overcoming viral immune evasion strategies. Blood. (2011) 117:848–56. 10.1182/blood-2010-08-301374 21030563

[243] Shemer-Avni Y Kundu K Shemesh A Brusilovsky M Yossef R Meshesha M. Expression of NKp46 splice variants in nasal lavage following respiratory viral infection: domain 1-negative isoforms predominate and manifest higher activity. Front Immunol. (2017) 8:161. 10.3389/fimmu.2017.00161 28261217 PMC5309248

[244] Pesce S Greppi M Tabellini G Rampinelli F Parolini S Olive D. Identification of a subset of human natural killer cells expressing high levels of programmed death 1: a phenotypic and functional characterization. J Allergy Clin Immunol. (2017) 139:335–46 e3. 10.1016/j.jaci.2016.04.025 27372564

